# Design and analysis of ELM-based predefined time sliding mode adaptive controller for PMLM position control under physical constraints

**DOI:** 10.1038/s41598-024-55444-4

**Published:** 2024-03-05

**Authors:** Saleem Riaz, Bingqiang Li, Rong Qi

**Affiliations:** School of Automation, Northwestern Polytechnical University, Xi’an, 710072 China

**Keywords:** Permanent magnet linear motor (PMLM), Predefined time convergence (PDTC), Control saturation, Sliding mode control (SMC), Extreme learning machine (ELM), Engineering, Electrical and electronic engineering

## Abstract

Achieving accurate position tracking for robotics and industrial servo systems is an extremely challenging task, particularly when dealing with control saturation, parameter perturbation, and external disturbance. To address these challenges, a predefined time convergent sliding mode adaptive controller (PTCSMAC) has been proposed for a permanent magnet linear motor (PMLM). A novel sliding mode surface (SMS) with predefined time convergence PDTC has been constructed, which ensures that the error converges to zero within the prescribed time. The system not only meets the expected performance standards but also has a uniformly bounded motor speed. The trajectory tracking error in SMS is proven to converge to zero within the predefined time. This predefined time stability of the closed-loop system has been demonstrated by using the Lyapunov stability criterion with PDTC. The convergence time (CT) can be arbitrarily set, and the upper bound of it is not affected by the initial value and control parameters of the system. A new updated version of extreme learning machine (ELM) is introduced to approximate the uncertain part of the system based on PDTC. The ELM is also provided with the hyperbolic tangent function to estimate the saturation constraint. This is done by converting the function into a linear function concerning the unconstrained control input variable. Then, based on established stability, a novel sliding mode adaptive controller (PTCSMAC) with predefined time convergence is designed. The convergence time (CT) of the controller is unaffected by the initial conditions as well as the control parameters. The rigorous numerical simulations on the PMLM model with complex disturbances verify the strong robustness and high-precision tracking characteristic of the proposed control law.

## Introduction

High precision control and stable speed of the linear motion system directly affect the processing and positioning accuracy of high-end equipment, industrial robotics^1,2^ and position control automation. The PMLM motor is excited by the permanent neodymium (NdFeB) magnet that can improve the feed speed in a short time and has the advantages of high positioning accuracy, high thrust density, and high efficiency. It is widely used in high-grade CNC machine tools^3^, lithography machines, rail transit and other high-precision motion control fields. However, factors such as external load disturbance, mechanical friction disturbance, unmodeled dynamics and time-varying uncertainties will directly act on PMLM. At the same time, PMLM is limited by mechanical design, and there are physical constraints of bounded input and output, which greatly increases the difficulty of controlling PMLM. It is a challenging task to track the position precisely, response speed and anti-disturbance of the PMLM system and meet the requirements of its application; it is necessary to do further research on its control mode.

Many control algorithms are used in the control design of PMLM motors. Traditional PID control has defects such as an unsatisfactory effect on time-varying uncertainty suppression and low control accuracy. Scholars use neural networks or fuzzy systems to improve the classical PID, which effectively improves the robustness and control accuracy of PMLM. As shown in Article^4^, a backpropagation (BP) neural network is utilized to recognize the uncertain part in the permanent magnet synchronous motor system online, and PID control parameters are self-tuning. In paper^5,6^, a fuzzy system is used to adjust PID control gain online, which improves the anti-interference of the PMLM synchronous motor system. In view of the high coupling, nonlinearity and uncertainty of PMLM, nonlinear control methods such as inversion control, SMC^7^, active disturbance rejection control^8^, neural network (NN) control^9^ and fuzzy control^10^ are used in the design of position tracking controller of PMLM, effectively improving the position tracking accuracy of PMLM. However, these methods can only make the position of PMLM better the tracking error asymptotically converges to zero. In the actual control, it is very important to improve the response ability of the motor, so that a control method that can guarantee the faster response. In order to improve position tracking speed, terminal SMC theory is applied to position tracking control of PMLM to ensure that the error converges to zero within a finite time^11^. Although finite time control can accelerate the convergence speed of position tracking, the CT is related to the initial value of the system and the control parameters, making the CT uncontrollable^12^. Therefore, scholars put forward the fixed time control theory that the upper bound of the CT does not depends the initial value of the system^13,14^, so that the CT is associated with the controller parameters, which efficiently advances the controllability of the system. The control parameters determine the stability of the system, and the CT of fixed time is related to the control parameters, so the CT of the system is still difficult to be preset in advance, and it is difficult to meet some engineering applications that need to converge within the Prescribed time. Therefore, this paper will study the convergence theory of prescribed time, and give a range whose upper bound of CT does not rely on the control parameters and the initial value of the system The Lyapunov stability criterion is collected, and a robust controller with prescribed time convergence is designed, so that the trajectory tracking time of PMLM system can be preset in advance.

There are tremendous advancements in control theory that have seen the emergence of the prescribed time control methodology as a solution to previous design challenges. This method, initially proposed by Wang and Song^15,16^, is notable for its ability to achieve a predetermined settling time that is independent of both initial states and control parameters. However, the complexity of its design process becomes a hurdle, especially for high-order systems. Wang and Liang^17^ further refined this concept with the introduction of the practically prescribed time control. Yet, this adaptation did not effectively address the issue of control input singularity. Due to the limitation of practical engineering application environment, the PMLM motor has the constraint of control input saturation and position tracking trajectory bounded. Simultaneously, in order to make the position tracking error have better transient performance, the system parameters can be set in advance to make the motor position tracking error change within the preset performance function. In literature^18^, based on the prescribed performance control method, the controller has been constructed for the uncertain nonlinear system with full state constraints. Literature^19^ combined adaptive control technology with prescribed performance control theory to design an output feedback FTC for nonlinear systems based on neural network.

In order to address the PMLM motor control issue, several nonlinear control approaches for instance terminal sliding mode control (TSMC) and nonsingular terminal SMC (NTSMC) have been implemented^20^. In^21^, for instance, a fast NTSMC compensating control approach with periodic updates is presented. By determining the PMLM linear and nonlinear characteristics, the authors of^20^ were able to develop a more effective control system for PMLM motors precise motion control. In^22^, a radial-basis function-network-based intelligent SMC controller was developed to mitigate the impact of uncertainties and disturbances. There is a special attention given in recent years on the development of numerous kinds of discrete-time SMC rules, as a growing number of controllers based on digital computers are put into operation. For instance, control laws for the inequality constraints were developed in^23,24^, while fixed-time SMC controls were investigated in. Over the last few years, there has been a rise in interest in the discrete-time terminal sliding mode control (TSMC). In particular^25,26^, and^27^ offered information on the impact of fixed time SMC control and fast TSMC, while^28,29^ recommended a design of discrete-time TSMC. To recapitulate, there are very less findings that have been published, however, on the predefined time sliding mode control (PDTSMC) and its problem of robustness have been considered in this study. Above-mentioned study has almost ignored the parametric perturbation and input control constraints problem for PMLM motor. External disturbances such that extra load torque that are both ignored and predefined settling time are considered to be a characteristic of the initial states during the ongoing investigation of predefined stability. In addition, the additive sensitivity of the convergence rate pattern requires a considerable control strategy. Our latest research shows that the predetermined time convergence for nonlinear systems with undetermined initial conditions that is still an open problem.

The control algorithm based on performance constraint function can effectively ensure that the tracking error of the system trajectory is bounded, and the performance function changes in advance, but the derivative of the tracking error cannot be bounded. In order to solve the control input saturation constraint, scholars initially used sign function or saturation function to limit the control input amplitude, which would lead to chattering of the control input. Then, experts use the first or second order constrained differential equation to limit the amplitude of the control input to solve the chattering problem of the control input. The above saturation control strategies first design the controller without considering the control saturation constraint conditions, and then carry out post-limiting on the control input. Due to post-limiting, the actual input energy of the control strategy will be insufficient, resulting in divergence of the system. The theoretical analysis shows that the main reason is that the control input after limiting the amplitude is not equal to the control input to ensure the stability of the closed-loop system. For the purpose of theoretical insurance that the control torque after limiting amplitude can stabilize the closed-loop system, the control saturation constraint must be considered in the controller design. However, the nonlinear constraint of control saturation will increase the difficulty of controller design. Therefore, this paper adopts the hyperbolic tangent function to approximate control saturation constraint, and adopts the mean value theorem to convert the nonlinear constraint of control input into the control input offline functions simplify controller design. Based on the above analysis for the actual operation of PMLM motor this paper considers the influence of external load disturbance, mechanical friction disturbance, pulse dynamics and time-varying uncertainties in motor as well as the control input constraints. The position and velocity errors have bounded physical constraints in the application environment for which the CT a sliding mode adaptive control algorithm is designed to track the output trajectory accurately.

The innovative key features and main contributions of this paper are as follows:

1. A prescribed time convergence Lyapunov stability criterion is given, which is independent of the control parameters and the initial value of the state. A novel SMS with prescribed time convergence is designed, and the position and velocity errors in the SMS are bounded. The hyperbolic tangent function is used to estimate the saturation constraint, and the hyperbolic tangent function is converted into a linear function about the input variables of unconstrained control, which makes the controller simplified and out of complexity.
2. A new sliding mode adaptive controller is designed based on the novel predefined time sliding surface where the trajectory tracking error (TTE) converges to the equilibrium point within the prescribed time. Proposed controller only needs to obtain the model error information of the controlled object. Due to mentioned adaptive characteristics we conclude that our proposed controller belongs to an adaptive family, which effectively advances the robustness of the controller.
3. The modified weight updated extreme learning machine (ELM) is used to approximate the uncertain package part of the system, so that the designed sliding mode adaptive controller only needs the displacement as well as velocity error information of PMLM system. Finally, we have demonstrated the various simulation results under various scenarios. Our proposed control law has been compared and verified with other numerous existing control strategies.

This paper is organized as follows: The problem formulation and control model description has been demonstrated in Section “Main target model description and problem formulation”. The detailed model having physical constraints as well as a sliding surface with a PDTC for tracking error convergence and the Lyapunov stability criterion have been described in Section “Description of disturbances in PMLM”. Furthermore, in the same Section “Description of disturbances in PMLM” the tracking error variance constraint as well as variation technique and the linearization of nonlinear inclusion criteria have been discussed. Section “PTCSMAC controller design” illustrates the design of the proposed PTCSMAC Controller based on ELM adaptive law. Simulation analysis for various cases and for the different initial conditions has been described in Section “Simulation verification”. The results and discussions are also given in the same Section “Simulation verification”. Overall conclusion of our proposed study is drawn in the end of this paper in Section “Conclusion”.

## Main target model description and problem formulation

The second order nonlinear model of PMLM motor is taken as:

1 $$\left\{ \begin{gathered} \dot{x}_{1} (t) = x_{2} (t) \hfill \\ \dot{x}_{2} (t) = - \frac{{k_{f} k_{e} }}{Rm}x_{2} (t) + \frac{{k_{f} }}{Rm}u(t) - \frac{F(t)}{m} \hfill \\ y(t) = x_{1} (t) \hfill \\ \end{gathered} \right.$$

where $x_{1} (t)$ is the displacement (mm), $x_{2} (t)$ is the angular velocity (mm/s), $u(t)$ represents input voltage (V),$R$ is the resistance (Ω), $m$ is the motor mass (kg), $k_{f}$ is the thrust constant, $k_{e}$ is the anti-electric force (Vs/rad), $F(t)$ is the external interference torque including the friction force $F_{{{\text{friction}}}} (t)$, pulse force $F_{{{\text{ripple}}}} (t)$ and load torque interference $F_{L} (t)$ (Nm), $y(t) \in {\mathbf{R}}^{m}$ is the output variable.

In this part we can take the following form of the PMLM model for the ease of generality of the theory. The dynamical model of PMLM is described as follows:

2 $$\left\{ \begin{gathered} \dot{x}_{1} (t) = x_{2} (t) \hfill \\ \dot{x}_{2} (t) = f(\theta ,{\mathbf{x}}(t),t) + Nu(t) + d(t) \hfill \\ y(t) = x_{1} (t) \hfill \\ \end{gathered} \right.$$

Now we can assumed and take the parametric perturbations such that $M = M_{0} + \Delta M$ and $N = N_{0} + \Delta N$ for which nominal parameters are $M_{0} = \frac{{k_{f} k_{e} }}{Rm},N_{0} = \frac{{k_{f} }}{Rm},d = \frac{F(t)}{m}$, then there is the whole system which has combined disturbances model is described in following way:

3 $$\left\{ \begin{gathered} \dot{x}_{1} (t) = x_{2} (t) \hfill \\ \dot{x}_{2} (t) = f(\theta ,x(t),t) + (N_{0} + \Delta N)u(t) + d(t) \hfill \\ y(t) = x_{1} (t) \hfill \\ \end{gathered} \right.$$

where ${\mathbf{x}}(t) = [x_{1} (t),x_{2} (t)]^{\rm T}$,$\theta (t)$ are state representation of variables and a combined nonlinearity respectively. And $f(\theta ,{\mathbf{x}}(t),t)$ here representing the smooth continuous functions. While the $u(t)$ is the control input and it should satisfy $|u(t)| \le u_{{\text{M}}}$. It is worth to note that $F(t)$ is a lumped disturbance that comprises frictional $F_{friction} \left( t \right)$, ripple $F_{{{\text{ripple}}}} (t)$ and load torque $F_{L} (t)$ disturbances.

### Control saturation constraint transformation

In order to solve the nonlinear bounded constraint problem of control input $u(t)$, a hyperbolic tangent function $tanh(t)$ with smooth, antisymmetric, monotone and uniformly bounded is used to replace the saturated constraint input $u(t)$. Based on the mean value theorem, the nonlinear bounded constraint in control saturation is transformed into a linear function about unbounded input $v(t)$.

According to the bounded constraints of we can take the form of controller input as: $||u(t)|| \le u_{M}$, where $u_{M}$ is the upper bound of $u(t)$, then respective design of the function $v(t)$ about $||u(t)|| \le u_{M}$ is as follows:

4 $$g(v(t)) = u_{{\text{M}}}\, tanh\left( {\frac{v(t)}{{u_{{\text{M}}} }}} \right)$$

Conferring to the property of the function $tanh(t)$, $||g(v(t))|| \le u_{{\text{M}}}$ is known. Therefore, $g(v(t))$ should have been used to approximate $u(t)$, and $v(t)$ is unconstrained. Thus, the design of saturated constrained input $u(t)$ is transformed into that of unconstrained input $v(t)$. Assume that the approximate error of $g(v(t))$ can be obtain by approximating $u(t)$ as follows:

5 $$\Delta u(t) = u(t) - g(v(t))$$

We know that $g(v(t))$ is bounded by the boundedness of $u(t)$ and the $\Delta u(t)$ boundedness of $|\Delta u(t)| \le b_{u}$. It is obvious that the control $u(t)$ is bounded as we take the bounded $g(v(t))$ and its corresponding value of $\Delta u(t)$. We can take the $\Delta u(t)$ as $|\Delta u(t)| \le b_{u}$.

The function $g(v)$ is continuously differentiable. By keeping in view, the mean value theorem, there exists $\eta$ between 0 and $v$, which makes $g(v) - g(0) = \dot{g}(\eta )(v - 0)$ valid. According to the definition of $tanh(t)$, $g(0) = 0$, then

6 $$g(v) = \dot{g}(\eta )v$$

It can be seen that $g(v)$ is represented as a linear function about unconstrained input $v(t)$ after this transformation, which effectively simplifies the design difficulty of controlling input $v(t)$. By taking the derivation of $g(v)$ with respect to $v$, and we will get $\dot{g}(\eta ) = u_{{\text{M}}} \frac{{\text{d}}}{{{\text{d}}v}}tanh(\frac{v}{{u_{{\text{M}}} }})|_{v = \eta } = sech^{2} (\frac{\eta }{{u_{{\text{M}}} }})$, so one can have the following result.

7 $$\dot{g}(\eta ) \ge 1$$

By synthesizing the above transformation, the system with control saturation constraints (3) can transform the model without control saturation constraints

8 $$\left\{ \begin{gathered} \dot{x}_{1} (t) = x_{2} (t) \hfill \\ \dot{x}_{2} (t) = f(\theta ,x(t),t) + N\dot{g}(\eta )v + N\Delta u(t) + d(t) \hfill \\ y(t) = x_{1} (t) \hfill \\ \end{gathered} \right.$$

### Prescribed performance function (PPF)

**Figure 1:**
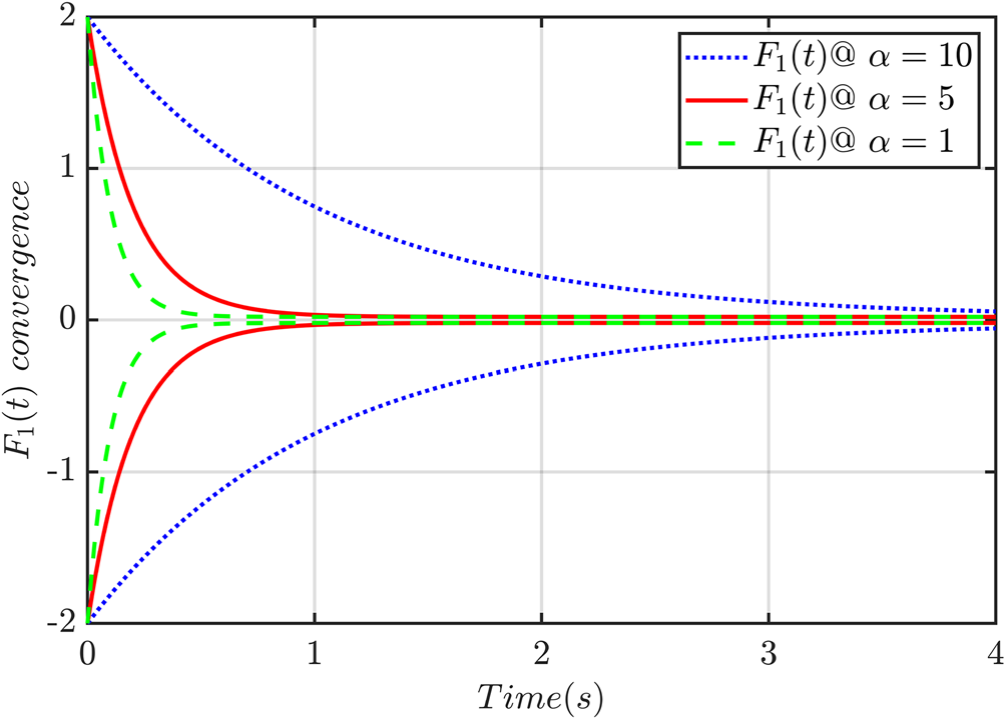
Convergence of transformation function for different values of $\alpha$.

The prescribed performance function (PPF) is generally adopted to design the controller. To ensure that the trajectory tracking error has better transient performance such as convergence rate, overshoot, and steady-state error in the whole control process. There is a trajectory tracking error ${\mathbf{e}}(t)$, that should meet the constraints such that $- \underline {\delta } F_{1} (t) < e_{i} (t) < \overline{\delta }F_{1} (t)$, for which the parameters $\underline {\delta }$ and $\overline{\delta }$ should be taken carefully according to the practical needs. Suppose the expected trajectory $y_{{\text{d}}} (t)$ of the nonlinear system (8) is, then the prescribed performance function (PPF) of TTE $e(t) = y(t) - y_{{\text{d}}} (t)$ is:

9 $$F_{1} (t) = (\rho_{0} - \rho_{\infty } )e^{ - \alpha t} + \rho_{\infty }$$

where $\rho_{0} > e(0),\rho_{0} > \rho_{\infty } \ge 0$ is the design parameter, $\rho_{0}$ is the initial value, $\rho_{\infty }$ signifies its steady-state cost of said function, determines the final steady-state interval of the performance function and the $\alpha$ is its convergence rate. The performance of the prescribed function for different values of convergence rate is illustrated in Fig. 1.

The condition for the creation of the PPF is that the initial value Fig. 1 of the trajectory tracking error must meet $|e_{i} (0)| < \rho_{0}$, otherwise, the controller will fail, resulting in the parameter $\rho_{0}$ of the PPF being heavily dependent on the initial value $e_{i} (0)$ of the trajectory tracking error. The state initial value of some systems is unknown and even changes the expected trajectory according to the actual application requirements, so that the initial value $e_{i} (0)$ of the system trajectory tracking error cannot be obtained in advance, restricting the practical application of the PPF. To solve the setting problem of the parameter $\rho_{0}$ in the PPF which is influenced by the initial value of system trajectory tracking error, and expand the application range of the PPF, this paper adopts the error conversion function $\xi (t)$ with monotonically decreasing characteristics to convert the trajectory tracking error ${\mathbf{e}}(t)$ into a new error variable $\xi (t)$ whose initial value at the origin.

### Main control objective

Main key point is to design the control input $u(t)$ for the nonlinear system with parameter perturbation, external interference, control saturation and bounded tracking error performance constraints (8), so that the system output $y(t)$ accurately tracks the expected trajectory $y_{{\text{d}}} (t)$ within a prescribed time $T_{s}$, and the TTE $e(t)$ meets the performance constraints $|e(t)| < F_{1} (t)$, bounded tracking error derivative.

## Description of disturbances in PMLM

**Figure 2:**
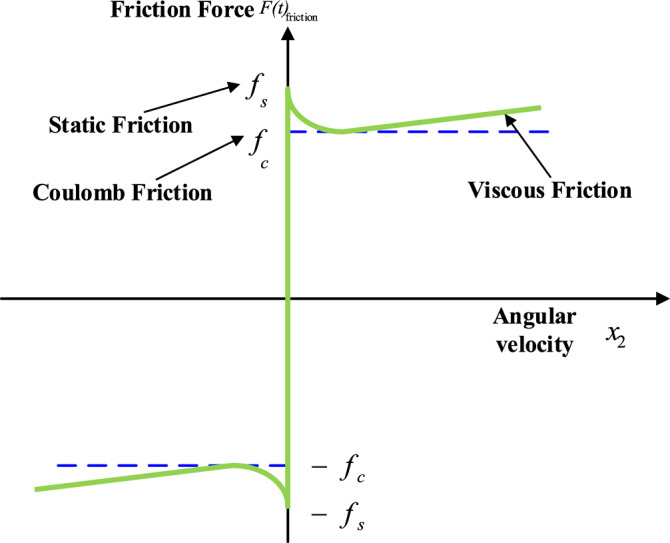
Stribeck friction curve.

Stribeck effect is also called stribeck friction that means a phenomenon which is observed in PMLM and other sliding mode control systems. This can also be described the variation in frictional behavior at different velocities of relative motion between two surfaces in contact. It is obvious that a PMLM system contains two primary categories of friction: a static or a Coulomb friction and a dynamic or a viscous friction. The stribeck curve is described as when the relative velocity between the permanent magnet and the stator changes, the frictional behavior follows a specific pattern. A typical stribeck curve characteristically comprises of three regions: High-Velocity Region: In this region at high relative velocities, the frictional force is mainly governed by the dynamic friction or viscous friction. Also, a frictional force remains relatively constant with changes in relative velocity. Mixed-Friction Region: In this region as the velocity decreases, the frictional force transitions from being dominated by dynamic friction to a combination of both static and dynamic friction. Such type of transition region is known as the mixed-friction region. Low-Velocity Region: In this specific region a very low relative velocities, the frictional force is primarily influenced by static friction. Additionally, the frictional force increases significantly with decreasing velocity, and ultimately the stribeck curve exhibits a steep rise. The model for friction $F_{{{\text{friction}}}} (t)$ is show as in the following Fig. 2. The stribeck curve is famous technique for describing friction. It shows the various friction values and the force of friction and velocity relate to one another. Equation (2) represents the stribeck friction. We can now describe the condition for which if static friction is $\left| {x_{2} (t)} \right| < \alpha$ and the threshold value is taken as $\alpha$, a dynamic friction $F_{friction} \left( t \right)$ then written as:

$$F_{{{\text{friction}}}} (t) = \left\{ {\begin{array}{*{20}c} {f_{m} \,\,\,\,\,\,\,\,\,\,\,\,\,\,\,\,\,\,\,\,\,\,\,\,\,f(t) > f_{m} } \\ {f(t)\,\,\,\,\,\,\,\,\,\,\,\,\,\,\, - f_{m} < f < f_{m} } \\ { - f_{m} \,\,\,\,\,\,\,\,\,\,\,\,\,\,\,\,\,\,\,\,\,\,\,\,\,f(t) < - f_{m} } \\ \end{array} } \right.$$

To understand a Stribeck friction effect is crucial in PMLM systems such as its performance, efficiency, and precision during operation. Researchers, engineers and designers should consider such kind of phenomenon while designing PMLM to ensure smooth operation and to minimize undesirable effects. For instance, a stiction (sticking) and hysteresis in the motion control system. Stribeck friction can be managed through proper lubrication and careful selection of materials which allows to optimize the performance of PMLM systems. If static friction $\left| {x_{2} (t)} \right| > \alpha$ then $F_{{{\text{friction}}}} (t)$ becomes:

10 $$F_{{{\text{friction}}}} (t) = \left[ {f_{c} + (f_{s} - f_{c} )e^{{ - (x_{2} /x_{2s} )^{2} }} + f_{v} x_{2} } \right]{\text{sgn}} (x_{2} )$$

From the above expression the parameter $f_{v}$ signifies coefficient for viscous friction, $x_{2s}$ is taken as the experimentally calculated parameter for lubrication, $f_{c}$ is smallest value of the Coulomb friction, and the static one is exhibited by $f_{s}$.

In a motor control system, frictional force refers to the resistance encountered by moving parts within the motor due to the contact and interaction between surfaces. Frictional forces can have both static and dynamic components, affecting the overall performance and efficiency of the motor.

Typically, PMLM motor comprises two primary types of frictional forces in its position control system: Static Friction: This is the force that must be overcome to set a stationary motor in motion and it is the initial resistance that needs to be surpassed before the motor starts rotating. Dynamic Friction: Once the motor is in motion, dynamic frictional forces act against the movement, causing resistance to the rotation of the components of the motor. Dynamic friction can reduce the efficiency of the motor and increase power losses. Frictional forces can be minimized by using high-quality bearings, lubrication, and proper maintenance of the motor components. Reducing friction is essential to improve the efficiency, extend its lifespan, and optimize its performance in a motor control system.

Rendering the tooth groove effect in a PMLM motor assembly, a pulsative force $F_{{{\text{ripple}}}} (t)$ produced by reluctance and it could be mathematically written as:

11 $$F_{{{\text{ripple}}}} (t) = \sum\limits_{i = 1}^{n} {A_{i} \sin (\omega_{i} x_{1} + \Phi_{i} )}$$

where $\omega_{i}$ is used to represent velocity and $A_{i}$ is denoting the amplitude. While $\Phi_{i}$ denotes the phase angle.

### Preliminaries and Important Lemmas

In order to simplify the stability analysis, we can describe the respective lemmas which are given in the following form.

#### Lemma 1

^30,31^ For $q > 0,a > 1,b > 0,$ and has $\frac{1}{a} + \frac{1}{b} = 1$.

12 $$xy \le \frac{{q^{a} }}{a}|x|^{a} + \frac{1}{{bq^{b} }}|y|^{b}$$

#### Lemma 2

^32^ Bernoulli inequality: for any $x \ge - 1$, if $q \ge 1$, then $(1 + x)^{q} \ge 1 + qx$; If $0 < q \le 1$, then $(1 + x)^{q} \le 1 + qx$.

#### Lemma 3

^33^ For $x_{i} \in R,i = 1,2, \ldots ,n$ and $l \in [0,1][0,1]$, the following relation is true:

13 $$\left( {\sum\limits_{i = 1}^{n} {|x_{i} |} } \right)^{l} \le \sum\limits_{i = 1}^{n} {|x_{i} |^{l} }$$

#### Lemma 4

^33^ For $x_{i} \in R,i = 1,2, \ldots ,n$ and $l \in [1,\infty ]$, the following expression is true:

14 $$n^{1 - l} \left( {\sum\limits_{i = 1}^{n} {|x_{i} |} } \right)^{l} \le \sum\limits_{i = 1}^{n} {|x_{i} |^{l} }$$

### Tracking error bounded constraint transformation

To realize the performance constraint of tracking error variation within the predefined performance function $F_{1} (t)$(9), the variable $\xi$ is introduced as

15 $$\xi = \frac{e(t)}{{\sqrt {F_{1}^{2} (t) - e_{{}}^{2} (t)} }}$$

There is $\xi$(when $|\xi | \le b_{\xi }$) is bounded i.e.

$$\xi^{2} \le b_{\xi }^{2} \Rightarrow e^{2} (t) \le \frac{{b_{\xi }^{2} }}{{1 + b_{\xi }^{2} }}F_{1}^{2} (t) \Rightarrow |e(t)| < F_{1} (t)$$

It shows that when variable $\xi$ is bounded, the TTE $|e(t)| < F_{1} (t)$ satisfies the constraint of bounded TTE in the control target.

Now we can take the derivative of $\xi$ with respect to time, and we get

16 $$\dot{\xi } = \Gamma \left( {x_{2} - \dot{x}_{d} - \frac{{e\dot{F}_{1} }}{{F_{1} }}} \right)$$

where $$\Gamma = \frac{{F_{1}^{2} }}{{\sqrt {(F_{1}^{2} (t) - e_{{}}^{2} (t))^{3} } }}$$.

### Predefined time convergent sliding surface

In order to enhance the robustness of the controller and ensure that the system output converges to the expected trajectory within a PDT, the following SMS with a prescribed time convergence characteristic is designed in this paper.

17 $$S = \dot{\xi } + \frac{1}{{T_{s1} }}\frac{{q({\text{sig}}\,tanh(\xi ))^{1 - 1/q} }}{{{\text{sech}}^{2} (\xi )}}$$

In which $T_{s1}$ is predefined time and the variables are defined as ${\text{(sig}}tanh(\xi ))^{q} = |tanh(\xi )|^{q} {\text{sign}}(tanh(\xi ))$. By considering lemma 1 we know that $q > 0$ the tracking error ${\mathbf{e}}(t)$ located on the sliding surface (17) that has the characteristic of convergence of prescribed time. The following is given in the form of a theorem, and it is proved theoretically.

#### Comparative simulation analysis of various sliding surfaces

Although finite-time control can accelerate the convergence speed of tracking, but the convergence time is related to the initial value of the system and the controller parameters. In contrast with predefined time convergence is independent of control parameters and initial system states.

Linear sliding mode surface: $S = \dot{e} + ce$.

Terminal sliding surface: $$\sigma (e) = \dot{e} + ce^{\alpha }$$, $$0 < \alpha < 1$$ or $$\sigma (e) = \dot{e} + c{\text{sig}}(e)^{\alpha }$$$$0 < \alpha < 1$$.

Non-singular terminal sliding surface: $\sigma (e) = e + c\dot{e}^{\beta }$, $1 < \beta < 2$ or $\sigma (e) = \dot{e} + c{\text{sig}}(e)^{\beta }$ $1 < \beta < 2$.

**Figure 3:**
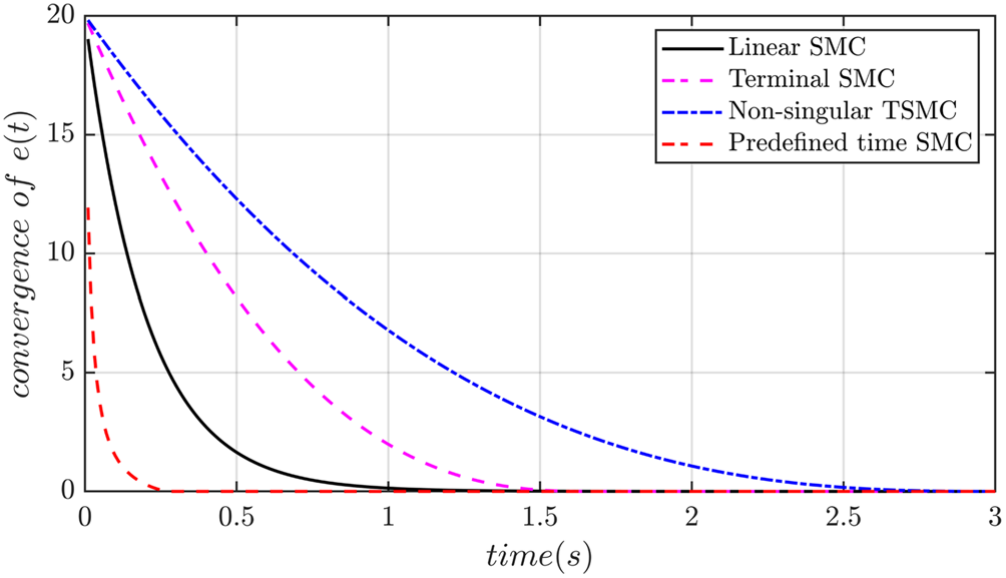
Comparison of different sliding surfaces.

From the perspective of convergence time, it can be seen that the tracking error in the linear sliding mode surface converges exponentially. The parameters determine the speed of tracking error convergence, but the convergence time tends to infinity. According to the Lyapunov stability criterion of finite-time convergence, the convergence time of tracking error in terminal and non-singular terminal sliding surface is finite-time convergence, and the convergence time is related to the initial value of tracking error and controller parameters. In order to analyze the convergence rate of tracking errors in each sliding mode plane, the control parameters for each surface are set as: TSMC: $\alpha = 0.6$, NTSMC: $\beta = {1}{\text{.6}}$, while $c = 5$ is chosen as the same for all the surfaces. It can be seen from the Fig. 3, we can analysis that under the same controller parameter Settings, the convergence time of the non-singular terminal sliding mode surface is the longest, and the convergence time of the predefined time is the shortest, mainly because the convergence time of the predefined convergence time in the sliding mode surface is set to 0.3 s.

The upper bound of tracking error convergence time in other sliding mode surfaces cannot be predefined in advance.

**Figure 4:**
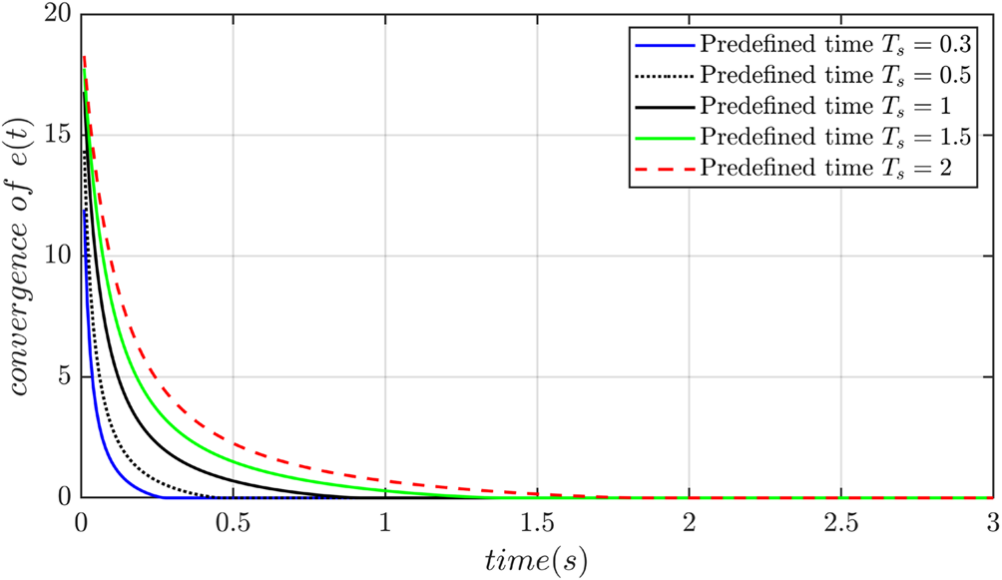
Proposed sliding surface performance for different predefined time settings.

In order to verify the validity of the predefined time convergence, the convergence time in the sliding mode surface of the predefined time convergence is randomly chosen a will, and the simulation result are depicted in the Fig. 4.

It is obvious that the convergence time of the tracking error in the sliding mode surface of predefined time convergence can be set arbitrarily according to the need of practical engineering applications. The actual convergence time of the tracking error can be ensured to be less than the predefined convergence time.

##### Theorem 1

For any prescribed time $T_{s1} > 0$, when the SMS (17) meets $S = 0$:

1. Let an initial tracking error is ${\mathbf{e}}(0) \ne 0$, ${\mathbf{e}}(t)$ it means we have to make it convergent which goes to zero within the prescribed time $T_{s1}$, and then we are able to say that CT also meets $t_{s}$:
  $$t_{s} = T_{s1} (tanh(\xi (0)))^{\frac{1}{q}} < T_{s1}$$
2. Suppose we have an initial value of the error which is ${\mathbf{e}}(0) = 0$, then respective time $t_{s} = 0$ at which ${\mathbf{e}}(t)$ converges to zero and also $S = 0$. So, it results as the TTE ${\mathbf{e}}(t) \equiv 0$.
3. The derivative $\dot{e}(t)$ of TTE is also bounded such that:
  $$|\dot{e}(t)| = \le \frac{{\sqrt[3]{{\rho_{0} }}}}{{\rho_{\infty }^{2} }}\frac{q}{{T_{s1} }} + \alpha (\rho_{0} - \rho_{\infty } )$$

##### Proof

For ease of proof, $\vartheta (\xi )$ is denoted as $\vartheta (\xi ) = tanh(\xi )$, then ${\text{sech}}^{2} (\xi ) = \dot{\vartheta }(\xi )$, so that the sliding surface (17) can be derived as follows:

18 $$S = \dot{\xi } + \frac{1}{{T_{s1} }}\frac{{q({\text{sig}}\vartheta (\xi ))^{1 - 1/q} }}{{\dot{\vartheta }(\xi )}}$$

From the Eq. (18) we can see that in order to avoid the singularity of the sliding surface $S$ the parameter $q$ should satisfy the condition $q > 1$. At $S = 0$, there is:

$$\begin{gathered} \dot{\xi } = - \frac{1}{{T_{s1} }}\frac{{q({\text{sig}}\vartheta (\xi ))^{1 - 1/q} }}{{\dot{\vartheta }(\xi )}} \Rightarrow \frac{1}{q}({\text{sig}}\vartheta (\xi ))^{{\frac{1}{q} - 1}} d\vartheta (\xi ) = - \frac{1}{{T_{s1} }}dt \hfill \\ \Rightarrow (\vartheta (\xi (t)))^{\frac{1}{q}} - (\vartheta (\xi (0)))^{\frac{1}{q}} = - \frac{1}{{T_{s1} }}t \hfill \\ \end{gathered}$$

Assuming there is that at time $t_{f}$, from the property of the combination $\xi (t_{f} ) = 0$, $\vartheta (\xi (t_{f} )) = 0$, $|tanh(\xi )| \le 1$ one can get:

19 $$\begin{gathered} (\vartheta (\xi (t_{f} )))^{\frac{1}{q}} - (\vartheta (\xi (0)))^{\frac{1}{q}} = - \frac{1}{{T_{s1} }}t_{f} \hfill \\ \Rightarrow t_{f} = T_{s1} (\vartheta (\xi (0)))^{\frac{1}{q}} \le T_{s1} \hfill \\ \end{gathered}$$

At $e(0) \ne 0$ as well as $\xi (0) \ne 0$ the convergence time $t_{f} = 0$ indicates that the sliding surface $S = 0$, which implies that the $tanh(\xi )$ obviously converges within the prescribed time $T_{s1}$. According to the monotonicity of $\xi (t)$ definitely approaches zero at the prescribed time $T_{s1}$ and the respective error $e(t)$ will also converges within $T_{s1}$. From Formula $q > 1$ (19), it can be seen that the smaller $q$ means the faster convergence time (CT) while the larger $q$ implies that the slower CT will be. But is noted that the upper bound of the convergence time is the prescribed time $T_{s1}$.

When $e(0) = 0$, $\xi (0) = 0$, the time $t_{f} = 0$ of $tanh(\xi )$ on the SMS (17)$S = 0$ converges to zero, indicating that the TTE $e(t)$ is identical to zero, specifically $e(t) \equiv 0$.

When $S = 0$, because ${\text{sech}}^{2} (\xi ) \ge 1$, $|tanh(\xi )| \le 1$, that is

20 $$|\dot{\xi }| = \frac{1}{{T_{s1} }}\frac{{q|({\text{sig}}\,tanh(\xi ))^{1 - 1/q} |}}{{{\text{sech}}^{2} (\xi )}} \le \frac{q}{{T_{s1} }}$$

From the associative formula (16) one can obtain the following expression.

$$|\dot{\xi }| = |\frac{{F_{1}^{2} }}{{\sqrt {(F_{1}^{2} (t) - e_{{}}^{2} (t))^{3} } }}\left( {x_{2} - \dot{x}_{{\text{d}}} - \frac{{e\dot{F}_{1} }}{F}} \right)| \le \frac{q}{{T_{s1} }}$$

And now we will obtain the following expression:

21 $$\Rightarrow |x_{2} - \dot{x}_{{\text{d}}} | \le \frac{{\sqrt {(F_{1}^{2} (t) - e_{{}}^{2} (t))^{3} } }}{{F_{1}^{2} }}\frac{q}{{T_{s1} }} + |\frac{{e\dot{F}_{1}^{{}} }}{F}| \le \frac{{\sqrt[3]{{\rho_{0} }}}}{{\rho_{\infty }^{2} }}\frac{q}{{T_{s1} }} + \alpha (\rho_{0}^{{}} - \rho_{\infty }^{{}} )$$

It indicates that the derivative of tracking error is bounded, and the upper bound is

$$|\dot{e}(t)| = |x_{2} - \dot{x}_{{\text{d}}} | \le \frac{{\sqrt[3]{{\rho_{0} }}}}{{\rho_{\infty }^{2} }}\frac{q}{{T_{s1} }} + \alpha (\rho_{0} - \rho_{\infty } )$$

According to theorem 1, when the SMS (17) satisfies $S = 0$, the TTE of the system not only obvious convergence within the prescribed time $T_{s1}$, but also makes the TTE $e(t)$ and its derivative $\dot{e}(t)$ both bounded.

### Lyapunov stability criterion for predefined time convergence

In this section we have designed a SMC with PDTC characteristics for nonlinear systems (8) and the theoretical analysis of prescribed time convergence, the Lyapunov stability criterion of prescribed time convergence is given in the form of a theorem and proved.

#### Theorem 2

For system (8), for any prescribed time $T_{s2} > 0$, the parameters $0 < p < 1,a > 0,b > 0$,$\Delta > 0$ if there is a Lyapunov function $V(t)$ which is radially unbounded and positively definite, satisfy

22 $$\dot{V}(t) \le \frac{ - \pi }{{2pT_{s2} \sqrt {ab} }}(aV^{1 - p} + bV^{1 + p} ) - \Delta$$

1. We have taken a stable system and it is globally stable as well in prescribed time, and the time $t_{s}$ converging to the equilibrium point at $V(0) \ne 0$ and the function $V(t)$ satisfy:
  $$\begin{gathered} t_{s} = \frac{{2T_{s2} }}{\pi }\arctan \left( {\sqrt{\frac{b}{a}} V^{p} (0)} \right) < T_{s2} \hfill \\ V(t) \le \left( {\sqrt{\frac{a}{b}} \tan \left( {\arctan \left( {\sqrt{\frac{b}{a}} V^{p} (0)} \right) - \frac{\pi }{{2T_{s2} }}t} \right)} \right)^{{{1 \mathord{\left/ {\vphantom {1 p}} \right. \kern-0pt} p}}} \hfill \\ \end{gathered}$$
2. The condition at which $V(0) = 0$, then $V(t) \equiv 0$ which indicates that the system state is always at the equilibrium point.

#### Proof

Suppose we have $\dot{V}(t) = \frac{ - \pi }{{2pT_{s2} \sqrt {ab} }}(aV^{1 - p} + bV^{1 + p} ) - \Delta$, so that

23 $$\frac{{{\text{d}}V}}{{{\text{d}}t}} = \frac{ - \pi }{{2pT_{s2} \sqrt {ab} }}aV^{1 - p} \left( {1 + \frac{b}{a}V^{2p} + \frac{{2pT_{s2} \sqrt {ab} }}{{\pi aV^{1 - p} }}\Delta } \right)$$

Transform Eq. (23) and differentiate

24 $$\frac{\pi }{{2T_{s2} }}{\text{d}}t = \frac{ - 1}{{1 + \left( {\sqrt{\frac{b}{a}} V^{p} } \right)^{2} + \frac{{2pT_{s2} \sqrt {ab} }}{{\pi aV^{1 - p} }}\Delta }}{\text{d}}\left( {\sqrt{\frac{b}{a}} V^{p} } \right)$$

Assume that we have $V(t_{s} ) = 0$ at time $t_{s}$, and we are now integrating the Eq. (24). Because $V \ge 0$, $\Delta \ge 0$ then $\frac{{2pT_{s2} \sqrt {ab} }}{{\pi aV^{1 - p} }}\Delta \ge 0$ we have:

$$\begin{aligned} \int_{0}^{{t_{s} }} {\frac{\pi }{{2T_{s2} }}{\text{d}}t} = - \int_{V(0)}^{{V(t_{s} )}} {\frac{{{\text{d}}\left( {\sqrt{\frac{b}{a}} V^{p} } \right)}}{{1 + \left( {\sqrt{\frac{b}{a}} V^{p} } \right)^{2} + \frac{{2pT_{s2} \sqrt {ab} }}{{\pi aV^{1 - p} }}\Delta }}} \le - \int_{V(0)}^{{V(t_{s} )}} {\frac{{{\text{d}}\left( {\sqrt{\frac{b}{a}} V^{p} } \right)}}{{1 + \left( {\sqrt{\frac{b}{a}} V^{p} } \right)^{2} }}} \\ = - \arctan \left( {\sqrt{\frac{b}{a}} V^{p} } \right)|_{V(0)}^{{V(t_{s} )}} \\ \Rightarrow & \frac{\pi }{{2T_{s2} }}t_{s} \le \arctan \left( {\sqrt{\frac{b}{a}} V^{p} (0)} \right) - \arctan \left( {\sqrt{\frac{b}{a}} V^{p} (t_{s} )} \right) \\ \Rightarrow & \arctan \left( {\sqrt{\frac{b}{a}} V^{p} (t_{s} )} \right) \le \arctan \left( {\sqrt{\frac{b}{a}} V^{p} (0)} \right) - \frac{\pi }{{2T_{s2} }}t_{s} \\ \Rightarrow & \sqrt{\frac{b}{a}} V^{p} (t_{s} ) \le \tan \left( {\arctan \left( {\sqrt{\frac{b}{a}} V^{p} (0)} \right) - \frac{\pi }{{2T_{s2} }}t} \right) \\ \Rightarrow & V^{p} (t_{s} ) \le \sqrt{\frac{a}{b}} \tan \left( {\arctan \left( {\sqrt{\frac{b}{a}} V^{p} (0)} \right) - \frac{\pi }{{2T_{s2} }}t} \right) \\ \Rightarrow & \left\{ \begin{gathered} V(t_{s} ) \le \left( {\sqrt{\frac{a}{b}} \tan \left( {\arctan \left( {\sqrt{\frac{b}{a}} V^{p} (0)} \right) - \frac{\pi }{{2T_{s2} }}t} \right)t_{s} } \right)^{{{1 \mathord{\left/ {\vphantom {1 p}} \right. \kern-0pt} p}}} \hfill \\ t_{s} \le T_{s2} \hfill \\ \end{gathered} \right. \\ \end{aligned}$$

#### Remark 1

The controller based on the given Lyapunov stability theory can ensure the state of the closed loop system converges to zero within the predefined time $T_{s2}$. Based on this Lyapunov stability criterion, the controller can guarantee the states of the closed loop system converges to neighborhood of the zero within the predefined time $T_{s2}$.

## PTCSMAC controller design

### Theorem 3

For system (8) for any prescribed time $T_{s1} > 0,T_{s2} > 0$, the control law is:

25 $$u(t) = u_{{\text{M}}} tanh\left( {\frac{v(t)}{{u_{{\text{M}}} }}} \right)$$

26 $$v(t) = - \frac{\pi }{{N\Gamma pT_{s2} \sqrt {ab} }}\left( {a\left( \frac{1}{2} \right)^{1 - p} {\text{sig}}S^{1 - 2p} + b\left( \frac{1}{2} \right)^{1 + p} {\text{sig}}S^{1 + 2p} } \right) - \frac{1}{N\Gamma }\left( {\frac{1}{{2\lambda^{2} }}S\hat{\theta }{\mathbf{h}}^{\rm T} {\mathbf{h}} + \frac{1}{2}S} \right)$$

27 $$\dot{\hat{\theta }} = \frac{1}{{2\lambda^{2} }}S^{2} {\mathbf{h}}^{\rm T} {\mathbf{h}} - \frac{\pi }{{pT_{s2} \sqrt {ab} }}\left( {\hat{\theta }^{{\frac{1}{1 + 2p}}} + \hat{\theta }^{{\frac{1}{1 - 2p}}} } \right)$$

The predefined time convergent sliding surface (17) is taken in the form of $S = \dot{\xi } + \frac{1}{{T_{s1} }}\frac{{q({\text{sig}}\,tanh(\xi ))^{1 - 1/q} }}{{{\text{sech}}^{2} (\xi )}}$, where the parameters can be chosen as $0 < p < 0.5$, $a > 0,b > 0,q > 0$ respectively.

1. System (8) will converge to the origin within the PDT $T_{s} = T_{s1} + T_{s2}$.
2. The tracking error $|e(t)| < F_{1} (t)$ and its derivative satisfy $|\dot{e}(t)| \le \frac{{\sqrt[3]{{\rho_{0} }}}}{{\rho_{\infty }^{2} }}\frac{q}{{T_{s1} }} + \alpha (\rho_{0} - \rho_{\infty } )$, control input also satisfies the condition $|u(t)| \le u_{{\text{M}}}$.

### Proof

Taking the derivative of the sliding surface $S$(17) with respect to time $t$ one can get.

28 $$\begin{aligned} \dot{S} = & \ddot{\xi } + \frac{q}{{T_{s1} }}\frac{{\text{d}}}{{{\text{d}}t}}\frac{{({\text{sig}}\vartheta (\xi ))^{1 - 1/q} }}{{\dot{\vartheta }(\xi )}} \\ = & \dot{\Gamma }\left( {x_{2} - \dot{x}_{{\text{d}}} - \frac{{e\dot{F}_{1}^{{}} }}{{F_{1} }}} \right) + \Gamma \left( {\dot{x}_{2} - \ddot{x}_{{\text{d}}} - \frac{{\text{d}}}{{{\text{d}}t}}\frac{{e\dot{F}_{1}^{{}} }}{{F_{1} }}} \right) + \frac{q}{{T_{s1} }}\frac{{\text{d}}}{{{\text{d}}t}}\frac{{({\text{sig}}\vartheta (\xi ))^{1 - 1/q} }}{{\dot{\vartheta }(\xi )}} \\ = & \dot{\Gamma }\left( {x_{2} - \dot{x}_{{\text{d}}} - \frac{{e\dot{F}_{1}^{{}} }}{{F_{1} }}} \right) + \Gamma \left( {f(\theta ,x(t),t) + N\dot{g}(\eta )v + N\Delta u(t) + d(t) - \ddot{x}_{{\text{d}}} - \frac{{\text{d}}}{{{\text{d}}t}}\frac{{e\dot{F}_{1}^{{}} }}{{F_{1} }}} \right) + \frac{q}{{T_{s1} }}\frac{{\text{d}}}{{{\text{d}}t}}\frac{{({\text{sig}}\vartheta (\xi ))^{1 - 1/q} }}{{\dot{\vartheta }(\xi )}} \\ = & \Gamma N\dot{g}(\eta )v + Q \\ \end{aligned}$$

**Figure 5:**
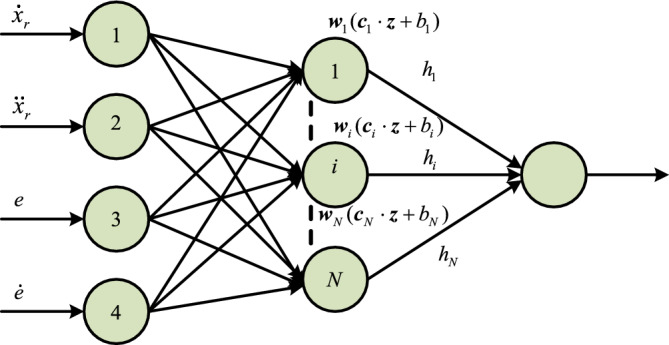
ELM Structure diagram.

In which

where $Q = \dot{\Gamma }\left( {x_{2} - \dot{x}_{{\text{d}}} - \frac{{e\dot{F}_{1} }}{{F_{1} }}} \right) + \Gamma \left( {f(\theta ,x(t),t) + N\Delta u(t) + d(t) - \ddot{x}_{{\text{d}}} - \frac{{\text{d}}}{{{\text{d}}t}}\frac{{e\dot{F}_{1} }}{{F_{1} }}} \right) + \frac{q}{{T_{{s1}} }}\frac{{\text{d}}}{{{\text{d}}t}}\frac{{({\text{sig}}\vartheta (\xi ))^{{1 - 1/q}} }}{{\dot{\vartheta }(\xi )}}$ is the encapsulation part, which contains the uncertain part of the system and relatively complex. This is the complete ELM package part, which contains the uncertain part of the system and more complex expressions. Extreme learning machine (ELM) model^34,35^, as shown in Fig. 5 will be used to approximate it.

Take the input ${\mathbf{z}} = (x_{2} ,e)^{\rm T}$ of the extreme learning machine and the output as the package part $Q$. The ELM can be regarded as a single hidden layer feedforward NN^36,37^ with $\tilde{N}$ nodes and the respective activation function is $h({\mathbf{x}})$. And then the mathematical description of ELM is as follows:

29 $$\sum\limits_{i = 1}^{{\tilde{N}}} {{\mathbf{w}}_{i} h_{i} ({\mathbf{c}}_{i} \cdot {\mathbf{z}},b_{i} )} = Q$$

where ${\mathbf{c}}_{i} = [c_{i1} ,c_{i2} , \ldots c_{in} ]^{\rm T} \in R^{n}$ is the $i$-th connection weight vector of the second hidden node and all n input nodes, $b_{i}$ is the node threshold, ${\mathbf{w}}_{i} = [w_{i1} ,w_{i2} , \ldots w_{im} ]^{\rm T} \in R^{m}$ is the connection weight vector of the second hidden node $i$ and all m output nodes, where ${\mathbf{c}}_{i} \cdot {\mathbf{z}}$ represents the inner product of vector ${\mathbf{c}}_{i}$ and vector ${\mathbf{z}}$. In the extreme learning machine, the connection weight ${\mathbf{c}}_{i}$ and node threshold $b_{i}$ are generated randomly, and ${\mathbf{w}}_{i}$ is the external weight vector to be identified. ELM cannot achieve accurate approximation to packaged part of $Q$, generally there is a bounded perturbation $\delta$, namely

30 $$Q = \sum\limits_{i = 1}^{{\tilde{N}}} {{\mathbf{w}}_{i}^{{}} h_{i} ({\mathbf{c}}_{i} {\mathbf{z}},b_{i} )} + \delta = {\mathbf{W}}^{\rm T} {\mathbf{h}}({\mathbf{z}}) + \delta$$

where ${\mathbf{h}}({\mathbf{z}}) = (h_{1} ({\mathbf{c}}_{1} {\mathbf{z}},b_{1} ), \ldots ,h_{{\tilde{N}}} ({\mathbf{c}}_{{\tilde{N}}} {\mathbf{z}},b_{{\tilde{N}}} ))^{\rm T}$,$|\delta | \le b_{Q}$.We have

31 $$SQ = S{\mathbf{W}}^{\rm T} {\mathbf{h}}({\mathbf{z}}) + S\delta \le \frac{1}{{2\lambda^{2} }}S^{2} \theta {\mathbf{h}}^{\rm T} {\mathbf{h}} + \frac{{\lambda^{2} }}{2} + \frac{1}{2}S^{2} + \frac{{b_{Q}^{2} }}{2}$$

where $\theta = ||{\mathbf{W}}||^{2}$. Because ${\mathbf{W}}$ is unknown so $\theta$ is also unknown. ${\mathbf{W}}$ is the outer weight vector of ELM, so $\theta$ is bounded, i.e.

32 $$0 < l_{\theta } < |\theta | \le b_{\theta }$$

In which, $l_{\theta }$ and $b_{\theta }$ indicate the upper and lower bounds of $\theta$, respectively. Let $\hat{\theta }$ be the estimate of $\theta$, and the estimated error be $\tilde{\theta } = \theta - \hat{\theta }$.

### Stability Proof

Construct the Lyapunov function $V_{1} = \frac{1}{2}S^{2}$ and take the derivative of $V_{1}$

33 $$\begin{aligned} & \dot{V}_{1} = S\dot{S} = S\Gamma N\dot{g}(\eta )v - SQ \\& \quad \le S\Gamma N\dot{g}(\eta )v - \frac{1}{{2\lambda^{2} }}S^{2} \theta {\mathbf{h}}^{\rm T} {\mathbf{h}} - \frac{1}{2}S^{2} - \frac{{\lambda^{2} }}{2} - \frac{{b_{Q}^{2} }}{2} \\ \end{aligned}$$

From Eq. (13)$\dot{g}(\eta ) \ge 1$, $- \dot{g}(\eta ) \le - 1$ is obtained. Substitute sliding mode adaptive controller (26) into Eq. (33), the following expression is obtained

34 $$\begin{aligned}& \dot{V}_{1} = - \frac{{\dot{g}(\eta )\pi }}{{pT_{s2} \sqrt {ab} }}\left( {a\left( \frac{1}{2} \right)^{1 - p} S^{2 - 2p} + b\left( \frac{1}{2} \right)^{1 + p} S^{2 + 2p} } \right) \\ & \qquad\quad- S\dot{g}(\eta )\left( {\frac{1}{{2\lambda^{2} }}S\hat{\theta }{\mathbf{h}}^{\rm T} {\mathbf{h}} + \frac{1}{2}S} \right) \\ &\qquad\quad- \frac{1}{{2\lambda^{2} }}S^{2} \theta {\mathbf{h}}^{\rm T} {\mathbf{h}} - \frac{1}{2}S^{2} - \frac{{\lambda^{2} }}{2} - \frac{{b_{Q}^{2} }}{2} \\ &\quad \le - \frac{\pi }{{pT_{s2} \sqrt {ab} }}(aV_{1}^{1 - p} + bV_{1}^{1 + p} ) - \left( {\frac{1}{{2\lambda^{2} }}S^{2} \hat{\theta }{\mathbf{h}}^{\rm T} {\mathbf{h}} + \frac{1}{2}S^{2} } \right) \\ &\qquad\quad- \frac{1}{{2\lambda^{2} }}S^{2} \theta {\mathbf{h}}^{\rm T} {\mathbf{h}} - \frac{1}{2}S^{2} - \frac{{\lambda^{2} }}{2} - \frac{{b_{Q}^{2} }}{2} \\ &\quad= \frac{ - \pi }{{pT_{s2} \sqrt {ab} }}(aV_{1}^{1 - p} + bV_{1}^{1 + p} ) - \frac{1}{{2\lambda^{2} }}S^{2} \tilde{\theta }{\mathbf{h}}^{\rm T} {\mathbf{h}} - \frac{{\lambda^{2} }}{2} - \frac{{b_{Q}^{2} }}{2} \\ \end{aligned}$$

Let $$V_{2} = V_{1} + \frac{1}{2}\tilde{\theta }^{2}$$, then

35 $$\begin{aligned} \dot{V}_{2}& \le - \frac{\pi }{{pT_{s2} \sqrt {ab} }}(aV_{1}^{1 - p} + bV_{1}^{1 + p} ) \\&\quad - \tilde{\theta }\left( {\frac{1}{{2\lambda^{2} }}S^{2} {\mathbf{h}}^{\rm T} {\mathbf{h}} + \dot{\hat{\theta }}} \right) - \frac{{\lambda^{2} }}{2} - \frac{{b_{Q}^{2} }}{2} \\ \end{aligned}$$

because

$$\begin{gathered} \tilde{\theta }\hat{\theta }^{{\frac{1}{1 + 2p}}} = \left( {\tilde{\theta }^{1 + 2p} \theta - \tilde{\theta }^{2 + 2p} } \right)^{{\frac{1}{1 + 2p}}} \le \hfill \\ \left( {\frac{2p + 1}{{2p + 2}}|\tilde{\theta }^{1 + 2p} |^{{\frac{2p + 2}{{2p + 1}}}} + \frac{1}{2p + 2}|\theta |^{2p + 2} - \tilde{\theta }^{2 + 2p} } \right)^{{\frac{1}{1 + 2p}}} \hfill \\ = \left( {\frac{1}{2p + 2}|\theta |^{2p + 2} \left( {1 - \left( {\frac{{\tilde{\theta }}}{\theta }} \right)^{2 + 2p} } \right)} \right)^{{\frac{1}{1 + 2p}}} \hfill \\ \le \left( {\frac{{\theta^{2p + 2} }}{2p + 2}} \right)^{{\frac{1}{1 + 2p}}} - \left( {\frac{{(2p + 2)^{{\frac{ - 1}{{1 + 2p}}}} }}{2p + 1}\frac{{\tilde{\theta }^{2 + 2p} }}{{\theta^{{\frac{2p(2p + 2)}{{2p + 1}}}} }}} \right) \hfill \\ \end{gathered}$$

It is bounded by $\theta$,let ${\rm K}_{1} = \left( {{{\theta^{2p + 2} } \mathord{\left/ {\vphantom {{\theta^{2p + 2} } {2p + 2}}} \right. \kern-0pt} {2p + 2}}} \right)^{{\frac{1}{1 + 2p}}}$, $\kappa_{1} = {{\left( {2^{1 + p} (2p + 2)^{{\frac{ - 1}{{1 + 2p}}}} } \right)} \mathord{\left/ {\vphantom {{\left( {2^{1 + p} (2p + 2)^{{\frac{ - 1}{{1 + 2p}}}} } \right)} {\left( {(2p + 1)b_{\theta }^{{\frac{2p(2p + 2)}{{2p + 1}}}} } \right)}}} \right. \kern-0pt} {\left( {(2p + 1)b_{\theta }^{{\frac{2p(2p + 2)}{{2p + 1}}}} } \right)}}$ we will have

36 $$\tilde{\theta }\hat{\theta }^{{\frac{1}{1 + 2p}}} \le {\rm K}_{1} - \kappa_{1} \left( {\frac{1}{2}\tilde{\theta }^{2} } \right)^{1 + p}$$

similarly, we have

37 $$\tilde{\theta }\hat{\theta }^{{\frac{1}{1 - 2p}}} \le {\rm K}_{2} - \kappa_{2} \left( {\frac{1}{2}\tilde{\theta }^{2} } \right)^{1 - p}$$

where $${\rm K}_{2} = {{(\theta^{2 - 2p} } \mathord{\left/ {\vphantom {{(\theta^{2 - 2p} } {(2 - 2p))}}} \right. \kern-0pt} {(2 - 2p))}}^{{\frac{1}{1 - 2p}}}$$, and $$\kappa_{2} = \left( {{{(2^{1 - p} (2 - 2p)^{{\frac{ - 1}{{1 - 2p}}}} )} \mathord{\left/ {\vphantom {{(2^{1 - p} (2 - 2p)^{{\frac{ - 1}{{1 - 2p}}}} )} {(1 - 2p)}}} \right. \kern-0pt} {(1 - 2p)}}} \right)l_{\theta }^{{\frac{2p(2 - 2p)}{{1 - 2p}}}}$$.

If Eqs. (27), (36) and (37) are substituted into Eq. (35), then the following stability can be obtained.

38 $$\begin{aligned} & \dot{V}_{2} \le - \frac{\pi }{{pT_{s2} \sqrt {ab} }}(aV_{1}^{1 - p} + bV_{1}^{1 + p} ) \\ & \qquad\quad- \frac{\pi }{{pT_{s2} \sqrt {ab} }}\tilde{\theta }(\hat{\theta }^{{\frac{1}{1 + 2p}}} - \hat{\theta }^{{\frac{1}{1 + 2p}}} ) - \frac{{\lambda^{2} }}{2} - \frac{{b_{Q}^{2} }}{2} \\& \qquad\le \frac{ - \pi }{{pT_{s2} \sqrt {ab} }}\left( \begin{gathered} aV_{1}^{1 - p} + bV_{1}^{1 + p} + \kappa_{1} \left( {\frac{1}{2}\tilde{\theta }^{2} } \right)^{1 + p} \hfill \\ + \kappa_{2} \left( {\frac{1}{2}\tilde{\theta }^{2} } \right)^{1 - p} \hfill \\ \end{gathered} \right) \\ &\qquad\quad- \frac{{\pi ({\rm K}_{1} + {\rm K}_{2} )}}{{pT_{s} \sqrt {ab} }} - \frac{{\lambda^{2} }}{2} - \frac{{b_{Q}^{2} }}{2} \\ &\qquad \le - \frac{\pi }{{2pT_{s2} \sqrt {a^{*} b^{*} } }}\left( {a^{*} V_{2}^{1 - p} + b^{*} V_{2}^{1 + p} } \right) - \Delta \\ \end{aligned}$$

where the parameters are described and taken as $\Delta = \frac{{\pi ({\rm K}_{1} + {\rm K}_{2} )}}{{pT_{s2} \sqrt {ab} }} + \frac{{\lambda^{2} + b_{Q}^{2} }}{2}$,$a^{*} = \max \{ a,2\kappa_{2} \}$, $b^{*} = \max 2^{ - p} \{ b,\kappa_{1} \}$ respectively.

#### Remark 2

Theorem 2 states that a feedback system could converge to a specific surface within a PDT $T_{s2}$ which is given for the proposed PTCSMAC (26). As it is described in Theorem1, that the tracking error must converges to origin within a PDT within the sliding surface (17) (i.e. $S = 0$). This can be achieved when PMLM states reached and stabilize in the sliding mode surface (17). Hence, the system has obvious convergence at the appropriate PDT ($T_{s} = T_{s1} + T_{s2}$). We can conclude form Theorem1 proof that the error its derivatives are bounded.

## Simulation verification

### PMLM motor simulation parameters and controller description

**Figure 6:**
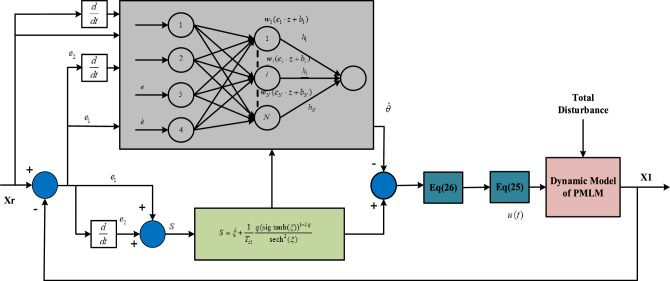
Overall Predefined time SMC via ELM control diagram.

The appropriate PMLM model parameters are specified as stated in Table 1 when using the mathematical model of PMLM as the simulation target. A complete PDTC sliding mode adaptive controller (PTCSMAC) structure is shown in the following Fig. 6 in order to emulate the PMLM motor model.

**Table 1:** PMLM model parameters.

Parameters	Symbols	Values	Units
Mass of motor	$m$	5.4	Kg
Force of resistance	$R$	*16.8*	Ω
Constant (force)	$k_{f}$	*130*	/
Back EMF	$k_{e}$	*123*	V/(rad/s)
Friction force	$f_{c}$	*10*	N
	$f_{s}$	*20*	
	$f_{v}$	*10*	
	$x_{2s}$	*0.1*	
Ripple force	$A_{1}$	*8.5*	N
	$\omega_{1}$	*314*	
	$A_{2}$	*4.25*	
	$\omega_{2}$	*314*	
	$A_{3}$	*2*	
	$\omega_{3}$	*314*	
Load disturbance	$F_{L}$	*If* *t* = 5, then, $F_{L} = 10\;{\text{Nm}}$	Nm

Significant values are in [italics].

The PMLM mode parameters with the symbolic descriptions are given in the following Table 1.

A PTCSMAC is an adaptive controller designed and it is used for control, and the respective parameters are taken correspondingly for high precision and accurate tracking of PMLM position. The following numerical values of all these parameters are: simulation time is 10 s, the initial value is $x_{1} (0) = 0.5,\;x_{2} (0) = 0.5,\;\hat{\theta }(0) = 0.5$ and the parameter of the performance function set as $\alpha$ = 0.05; $\rho_{0}$ = 2; $\rho_{\infty }$ = 0.003, the upper bound of the control saturation constraint is chosen different in different cases, the ELM activation function is selected as $h_{i} (z) = (1 + e^{{ - {\mathbf{zc}}_{i} + b_{i} }} )^{ - 1}$.

### Comparative simulation analysis under different predefined convergence times

*Case 1*: Step signal tracking performance

In this section first we can define all the parameters for controller and PMLM model are taken according to Tables 1 and 2 respectively. And predefined convergence time is set as shown in Table 2. The following numerical values of all these parameters are mentioned: total simulation time is 10 s, the initial value is $x_{1} (0) = 0.5,x_{2} (0) = 0.5,\;\hat{\theta }(0) = 0.5$ and the parameter of the performance function is $\alpha$ = 0.05; $\rho_{0}$ = 1; $\rho_{\infty }$ = 0.002, the upper bound of the control saturation constraint that is $u_{M}$ = 50 is not considered in this section, the parameters of the sliding variables have been chosen as: ${\text{q}} = 1000,T_{s1} = 1s$, the relevant parameters of the adaptive controller are, $T_{s2} = 1{\text{s}}$, p = 0.3, a = 100, b = 1.5, $\lambda$ = 0.30 the ELM activation function is selected as $h_{i} (z) = (1 + e^{{ - {\mathbf{zc}}_{i} + b_{i} }} )^{ - 1}$, and the reference input of the motor is $x_{r} = 1$.

**Figure 7:**
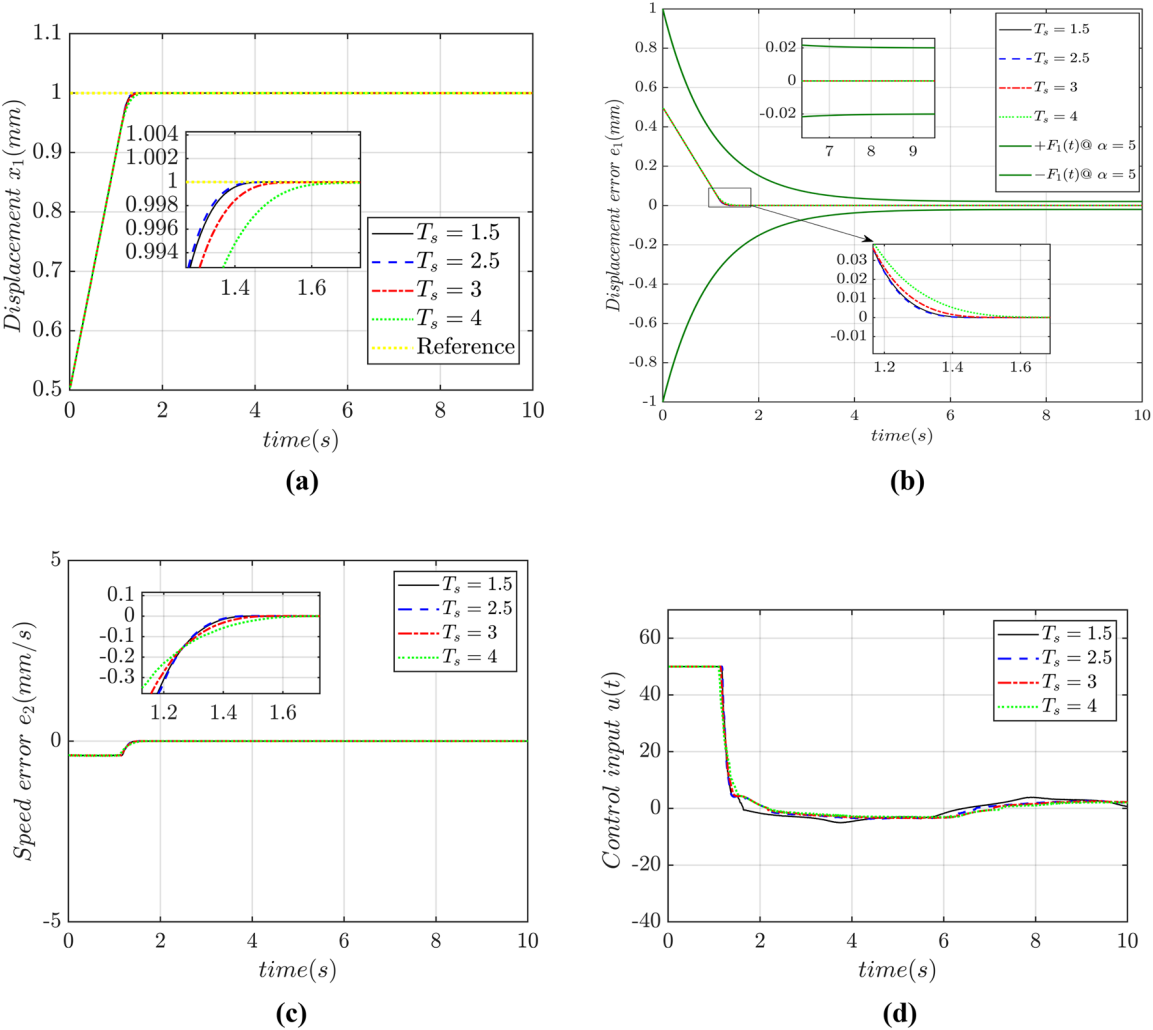
(**a**) Performance of displacement tracking, (**b**) Displacement tracking error, (**c**) Speed tracking error, (**d**) Control input.

Numerical simulation results show that:

1. The adaptive controller with predefined convergence time has strong robustness to parameter perturbation and external disturbance. Simulation results portrays that under the conditions of time-varying parameter perturbation, friction and time-varying load, the displacement of PMLM can accurately track the reference signal before the predefined convergence time based on the predefined time-convergence adaptive criteria as proposed in this research. It shows that the proposed adaptive controller has strong disturbance tolerant capability to the control saturations and strong robustness even having the internal parameter perturbations and external disturbances.
2. The displacement of PMLM can converge to the reference signal within a PDT. Figure 7a and b show that, when controller parameters are unchanged and the predefined convergence time is changed, under the condition of parameter perturbation, friction and time-varying load, the actual displacements of PMLM can accurately track reference signals before the PDTC, indicating that the displacement tracking convergence time of PMLM motor is not affected by controller parameters and initial state value of system, depends only on the PDTC. At the same time, the predefined convergence time is the upper bound of the CT of displacement tracking error, rather than the actual convergence time. Increasing the PDTC will increase the convergence time of PMLM displacement tracking error, but the convergence time of such error is less than 1.5 s, indicating that under the same controller parameters, although changing the PDT will affect the convergence rate of displacement TTE, the CT is not strictly in accordance with the PDTC.
3. Permanent magnet linear motor converges to the reference signal with better dynamic performance and higher tracking accuracy. Figure 7a and b show that under different predefined convergence times, the displacement tracking error of the motor can converge to zero with a high accuracy, with the accuracy reaching nano levels. The smaller the predefined convergence time is, the higher the precision of the motor displacement tracking error is. When the predefined convergence time increases, the displacement tracking error accuracy of the motor will decrease, but it can still reach the order of nano level. It is highlighted that the proposed controller can ensure that the motor can track the reference signal with higher accuracy. When the predefined convergence time is 1.5 s then the displacement tracking error under other predefined convergence times converges smoothly to zero in the form of exponential convergence, showing good dynamic control performance. But on the other hand, it also shows that in the process of adaptive control of the motor, the predefined convergence time is not too small, and this CT is easy to lead to the overshoot of displacement tracking error. Figure 7c shows that the speed tracking convergence time of the motor will increase with the increase of the predefined convergence time, but the magnitude of the speed tracking error can still reach the magnitude of micro level, and the speed tracking convergence time is less than 1.5 s, indicating that the speed of the PMLM motor has high tracking accuracy and fast convergence speed based on the controller designed in this paper.
4. The input voltage decreases with the increase of the PDTC, and the input voltage do not have chattering. Figure 7d shows that when the predefined convergence time is 1.5 s, the maximum input voltage reaches 50 V; when the PDTC is 2.5 s, the maximum input voltage almost 10 V; and when the PDTC is 3 s, the maximum input voltage decreases by an order of magnitude 8 as the PDTC gradually increases. In the whole control process, the maximum value of the control input voltage appears at the initial moment, and as the motor displacement TTE converges to zero, the value of the control input voltage gradually decreases to less than 1 V, indicating that a smaller predefined convergence time will lead to a larger control input voltage, and increasing the predefined convergence time can reduce the value of the control input voltage. During the whole control process, the control input voltage has no any chattering phenomenon. When the motor displacement converges to the reference signal, the control input voltage is in a straight line, showing good control performance of the proposed controller.

**Table 2:** Predefined time settings.

Total time	Parameters setting
$T_{s} = 1.5$	$T_{s1} = 1,T_{s2} = 0.5$
$T_{s} = 2.5$	$T_{s1} = 1.5,T_{s2} = 1$
$T_{s} = 3$	$T_{s1} = 1.5,T_{s2} = 1.5$
$T_{s} = 4$	$T_{s1} = 2,T_{s2} = 2$

*Case 2*: Tracking performance for square-wave signal

In this section a square wave signal is taken as a reference trajectory.

**Figure 8:**
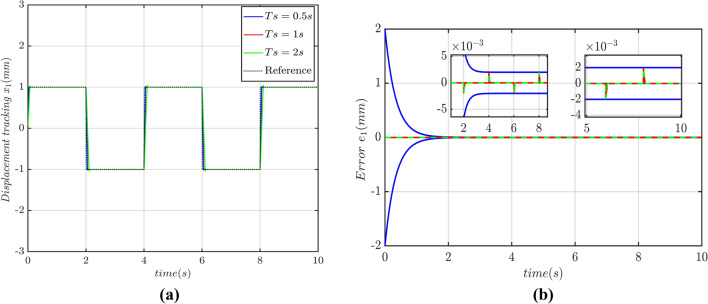
(**a**) Tracking of square wave signal, (**b**) displacement tracking error.

It is obvious that the displacement of PMLM fully tracks the reference square wave signal within a predefined time. The tracking is shown in Fig. 8a. It is worth noting that the controller parameters are remained same while the predefined convergence time is changed. It can be seen that the actual displacement perfectly track given reference signals before the PDTC under the parameter perturbation, friction and time-varying load which indicates that the displacement tracking convergence time of PMLM motor is not affected by controller parameters and initial state value of system. It is only dependent on the predefined time which can be set arbitrarily. It can be seen from Fig. 8b that predefined convergence time has upper bound of the CT of displacement tracking error, rather than the actual convergence time. By increasing the PDTC will ultimately increase in the convergence time of PMLM displacement tracking error. This convergence time of error is less than 1.5 s, indicating that changing the PDT will affect the convergence rate of displacement TTE but not the convergence time. Hence the proposed controller is robust and strong capability of error convergence within the predefined time.

*Case 3*: Tracking performance for sinusoidal signal

In this case we have taken a sinusoidal reference signal $x_{r} = \sin (t)$, and load is set as $F_{L} (t) = {\text{10sin(t)}}$. The simulation time is set to 10 s. While the initial values are set to $x_{1} (0) = 0.5,x_{2} (0) = 0.5,\;\hat{\theta }(0) = 0.5$ and the parameter of the performance function are $\alpha$ = 0.05;$\rho_{0}$ = 2;$\rho_{\infty }$ = 0.003. Control saturation constraint and its upper bound which is $u_{M}$ = 30 is not considered in this section. The sliding variables have been chosen as:${\text{q = }}500$, while other parameters of the adaptive controller are given as,p = 0.3,a = 1,b = 1.5,$\lambda$ = 0.30, The ELM activation function is selected as $h_{i} (z) = (1 + e^{{ - {\mathbf{zc}}_{i} + b_{i} }} )^{ - 1}$. The predefined convergence time settings are elaborated in the following Table 3.

**Table 3:** Predefined convergence time setting.

Total time	Parameters setting
$T_{s} = 0.2$	$T_{s1} = 0.1,T_{s2} = 0.1$
$T_{s} = 0.5$	$T_{s1} = 0.25,T_{s2} = 0.25$
$T_{s} = 1$	$T_{s1} = 0.5,T_{s2} = 0.5$
$T_{s} = 2$	$T_{s1} = 1,T_{s2} = 1$

**Figure 9:**
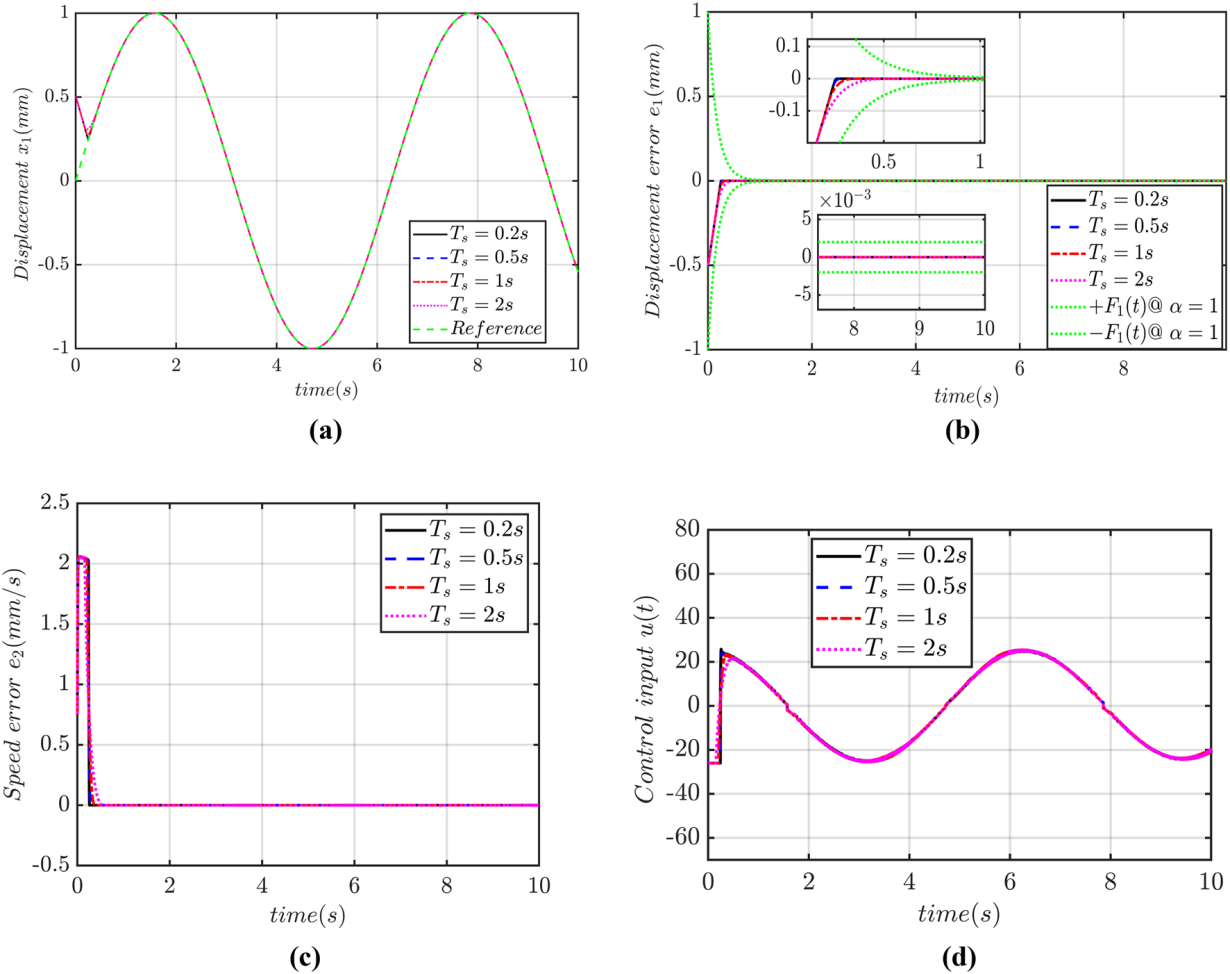
(**a**) Displacement tracking trajectory, (**b**) Displacement tracking error, (**c**) Speed tracking error, (**d**) Control input.

Figure 9a show that when the PDTC is changed, the proposed PTCSMAC can still ensure that the motor displacement can track the reference signal with high accuracy before the PDTC. The displacement tracking curve is shown in Fig. 9a. Figure 9b and c show that the convergence time of the displacement as well as speed errors which becomes larger as the PDTC increases. However, under the four predefined convergence times, the displacement and speed tracking error will converge to zero within 0.5 s. It can be witnessed that the displacement tracking error magnitude reaches up to nano level (see Fig. 9b) while the speed tracking error magnitude reaches micro level as shown in Fig. 9c. Displacement as well as speed tracking errors converge in the form of exponential convergence, and there is no overshoot in the whole process of convergence, which indicates that based on the proposed PTCSMAC. The precise tracking of displacement and speed of the motor can be ensured at a fast speed and has good dynamic performance, even if there are internal parameter perturbations and external disturbances in the system. Figure 7 shows that the smaller the predefined convergence time is, the larger the initial control input voltage will be. As the predefined convergence time increases, the initial control input voltage will decrease significantly as depicted in Fig. 9d. When the motor displacement tracking error tends to zero, the control input voltage will fluctuate with the change of the reference sine wave signal, but the control input voltage does not have chattering, which indicates that the proposed adaptive learning observer can effectively suppress the control input voltage chattering problem.

### Comparative simulation analysis of different controllers

#### Performance analysis under bounded control input constraints

To compare and analyze the effectiveness of PDT sliding mode adaptive controller (PTCSMAC), we have taken PID, Linear sliding mode controller (Linear SMC), and Nonsingular terminal sliding mode controller (NTSMC) and each controller structure has been described as follows:

$${\text{PID}}\;{\text{controller}}:u_{{{\text{PID}}}} = \Gamma_{p} e_{1} + \Gamma_{{\text{i}}} \smallint e_{1} {\text{d}}t + \Gamma_{{\text{d}}} e_{2}$$

$${\text{Linear}}\;{\text{SMC}}\;{\text{controller}}:\left\{ \begin{gathered} u_{{{\text{LSMC}}}} = \frac{1}{N}\left[ {\ddot{x}_{{\text{r}}} + Mx_{2} + \lambda e_{2} + \phi_{1} sat(s)} \right] \hfill \\ s = e_{2} + \psi e_{1} \hfill \\ \end{gathered} \right.$$

$${\text{NTSMC}}\;{\text{Controller}}:\left\{ \begin{gathered} u_{total} = u_{main} + u_{comp} \hfill \\ u_{main} = \frac{1}{N}\left( { - Me_{2} + M\dot{x}_{r} + \ddot{x}_{r} + F + \frac{1}{{\beta_{1} \upsilon_{1} }}\left| {e_{2} } \right|^{{2 - \upsilon_{1} }} sign\left( {e_{2} } \right) + \frac{{\beta_{2} }}{{\beta_{1} }}\left| {e_{2} } \right|^{{2 - \upsilon_{1} }} \left| {e_{1} } \right|^{{\upsilon_{1} - 1}} } \right) \hfill \\ u_{comp} = \frac{1}{N}\left[ {k_{1} s + k_{2} sign(s)} \right] \hfill \\ s = e_{1} + \beta_{1} \left| {e_{2} } \right|^{{\upsilon_{1} }} sign\left( {e_{2} } \right) + \beta_{2} \left| {e_{1} } \right|^{{\upsilon_{1} }} sign\left( {e_{1} } \right) \hfill \\ \end{gathered} \right.$$

In practical engineering applications, affected by the application environment, the actual control input voltage will have a bounded saturation constraint, that is, there is a maximum control input $u_{{\text{M}}}$, which requires the actual input $u$ to meet $|u| \le u_{{\text{M}}}$. The following is a comparative analysis of the effect of bounded control input constraints for different control techniques on the control performance of PMLM motor. In this part, we have considered that when the reference signal is a sinusoidal $x_{r} = \sin (t)$, the control parameters of each controller are given in Table 4, and only the control input is limited that is set as $u_{{\text{M}}} = 130$ v (maximum control input voltage).

**Table 4:** Parameters settings of different controller.

Controller		Parameter setting
PTCSMAC	Predefined time	$$T_{s1} = 0.5,T_{s2} = 0.5$$$${\text{q = }}2000$$
	Controller	p = 0.3, a = 100, b = 1.5,$\lambda$ = 0.30 $\alpha$ = 0.05;$\rho_{0}$ = 2;$\rho_{\infty }$ = 0.003
NTSMC	Sliding surface	$\beta_{1} = {1}{\text{.5}},\beta_{2} = {0}{\text{.5}},\upsilon_{1} = {1}{\text{.1}}$
	Controller	$k_{1} = 100,k_{2} = 1000$
PID	Controller	$k_{{\text{p}}} = 23000,k_{{\text{d}}} = 5000,k_{{\text{i}}} = 2$
Linear SMC	Sliding surface	$\psi = 100$
	Controller	$\phi_{1} = 200$

**Figure 10:**
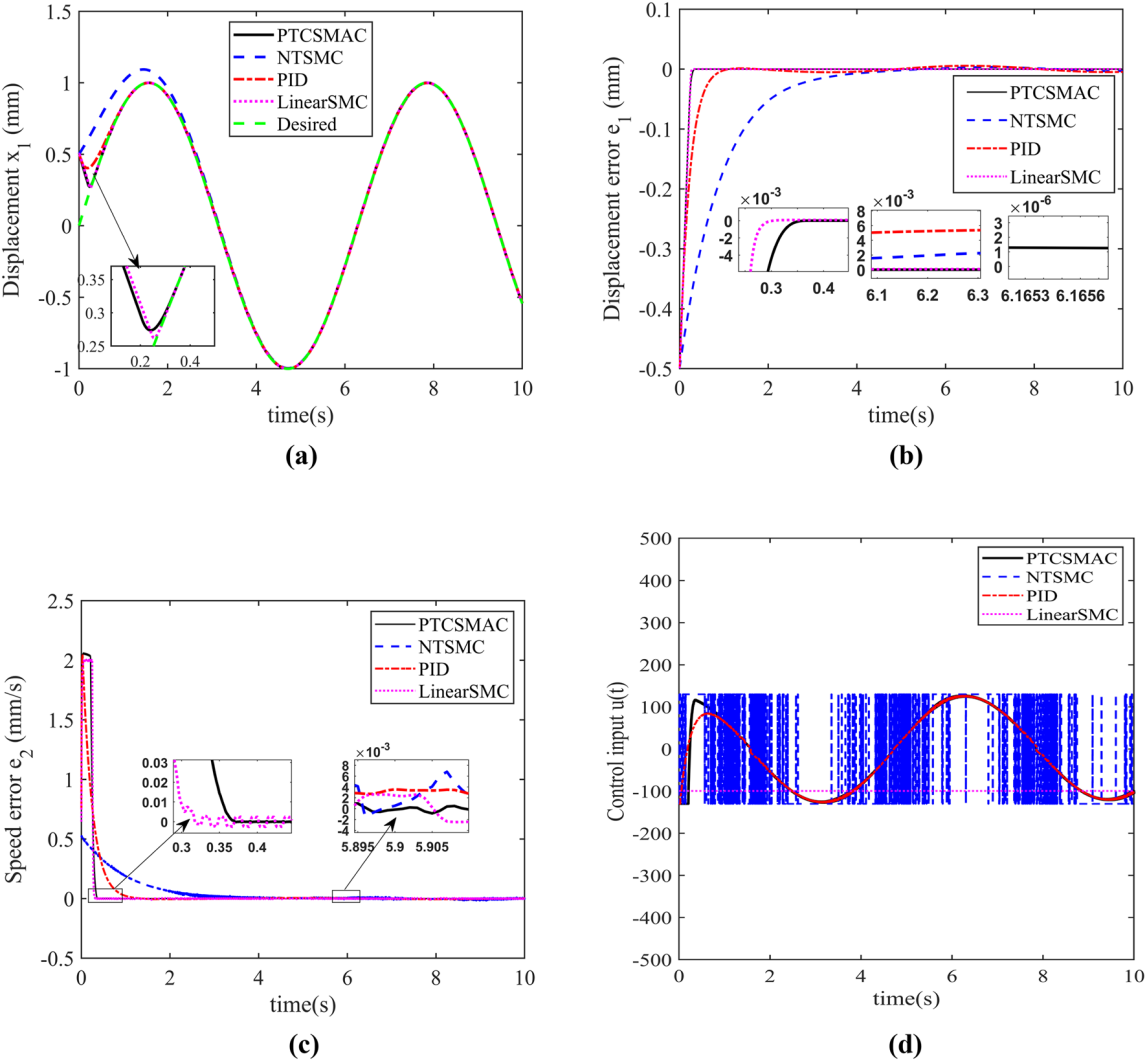
(**a**) Displacement tracking trajectory, (**b**) profile of the displacement tracking error, (**c**) Speed tracking error, (**d**) profile of the control input.

**Figure 11:**
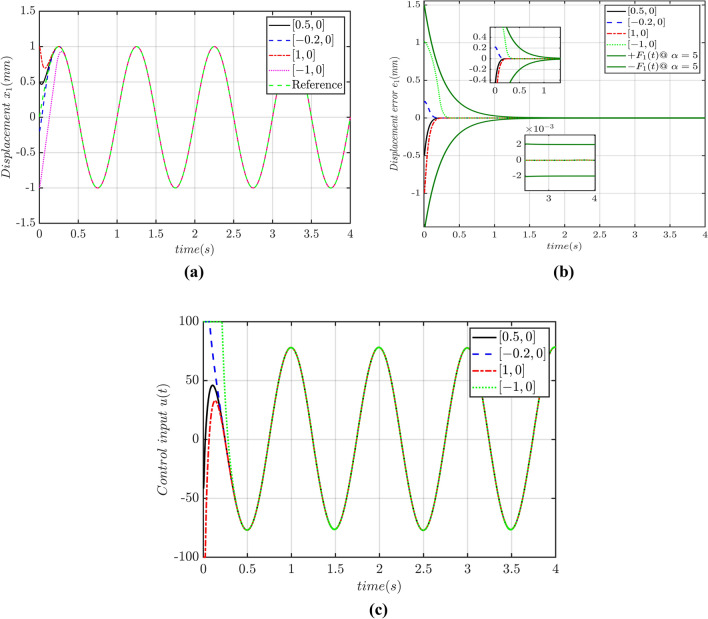
(**a**) Displacement tracking profile (**b**) tracking error profile (**c**) control input profile.

The numerical simulation outcome is exposed in the following figure (see Figs. 8, 9, 10 and 11).

The simulation results depicts that the four controllers can still realize the stability of the PMLM after the control input voltage is limited directly, so that the displacement of the motor converges to the reference sine wave signal, but the convergence time of the displacement is longer than that of the state of no control saturation constraint as shown in Fig. 10a. Among the four kinds of controllers, the convergence time of displacement tracking under the Linear SMC controller is the fastest, with a convergence time of 0.3 s, while this said time under the PTCSMAC controller is the second fastest, with an amount of 0.35 s. However, its convergence time still meets the requirement of predefined convergence time $T_{s} = 1$ s. Although the convergence time of the displacement in the Linear SMC controller is faster than that in the PTCSMAC controller, but the displacement tracking accuracy of the Linear SMC controller is not as high as that of the PTCSMAC controller as shown in Fig. 10b. Moreover, the steady-state value of the displacement tracking error of the Linear SMC controller as depicted in Fig. 10 is larger and there is oscillation. However, according to Fig. 10c our proposed PTCSMAC controller has smaller steady-state velocity tracking error and no oscillations. Figure 10d shows that among the four controllers, the control input voltage of NTSMC controller jumps up and down at the upper limit of the control input, and there is a large chattering phenomenon, while the control input voltage of the other three controllers is smooth without chattering. It can be concluded that our proposed control is robust, chattering free and more resilient to multiple uncertainties.

The above Fig. 11a shows the tracking performance of our proposed PTCSMAC controller under different initial conditions. The parameters of the controller remain same such as p = 0.3, a = 100, b = 1.5, $\rho_{\infty } = 0.002$, $\rho_{0} = 1.5$. The tracking performance of the displacement is precise regardless of the initial conditions. The upper and lower bounds of the prescribed performance function is also satisfied and error remains within the prescribed performance function as it is depicted in Fig. 11b. The overall control input is also satisfactory as shown in Fig. 11c. The rigorous simulation analysis shows that our proposed PTCSMAC controller is robust, chattering free and more resilient to load variations and input saturation as well as numerous uncertainties.

### Position tracking and error analysis for different activation function

In this section we can generally analyze the choice of different activation function for PMLM position control. The different activation functions can be chosen according to the user model and needs as described in Table 5.

**Table 5:** Different activation functions with mathematical clarification.

Choice of activation function	Mathematical description
Sigmoid Function (Sig F)	$G(a,b,x) = \frac{1}{{1 + e\,^{( - a \cdot x + b)} }}$
Hyperbolic Tangent Function (HTF)	$G(a,b,x) = \frac{{1 - e\,^{( - a \cdot x + b)} }}{{1 + e\,^{( - a \cdot x + b)} }}$
Hard Limit Function (HLF)	$$G(a,b,x) = \left\{ {\begin{array}{*{20}l} {1,{\text{ if a}}{\text{.x }} \le 0} \hfill \\ {0,{\text{ otherwise }}} \hfill \\ \end{array} } \right.$$
Cosine Fourier basis Function (CF cos)	$G(a,b,x) = {\text{Cos}} \,(a.x + b)$

**Figure 12:**
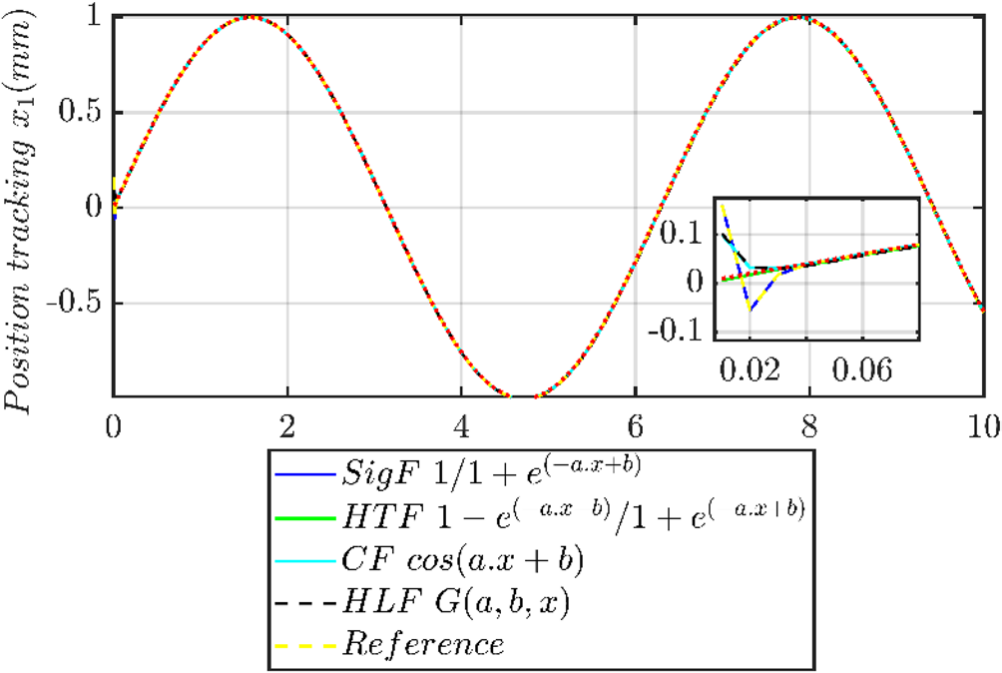
Position Tracking profile at different activation function.

From the Table 5 we can see that PMLM position tracking one can utilize various activation functions to determine the most accurate and efficient method for tracking positions in different scenarios. It is abvious that among these choices the Sigmoid Function (Sig F) is notable for its characteristic S-shaped curve, which provides a smooth and gradual transition between output values. The Hyperbolic Tangent Function (HTF) is often preferred due to its zero-centered nature, making it more efficient for gradient descent methods. While if one chooses the Hard Limit Function (HLF) for binary outputs which is useful in specific scenarios where a stark distinction is needed. When it comes to Cosine Fourier Basis Function (CF cos) it uses the cosine waves, valuable in representing periodic or oscillatory behaviors. As it can be seen from position tracking of PMLM (see Fig. 12), each of these functions has unique properties, making them more or less suitable for different types of position tracking tasks within PMLM position tracking systems.

The different errors expressions for the analysis are taken as: Integral square error (ISE), ${\text{ISE }} = \int e (t)^{2} \cdot dt$, Integral absolute error (IAE), ${\text{IAE }} = \int | e(t)| \cdot dt$, Integral time, absolute error (ITAE), ${\text{ITAE }} = \int t \cdot |e(t)|dt$, Integral time square error (ITSE), ${\text{ITSE }} = \int t \cdot \left[ {e(t)^{2} } \right] \cdot dt$ respectively. The particular simulations results has been given in the following Table 6 for each of the above mentioned error.

**Table 6:** Error results for different activation functions.

Activation Function	Sig F	HTF	CF cos	HLF
RMSE	2.00E−07	6.36E−07	0.0023	3.71E−06
IAE	0.08786	1.01442	1.01533	0.06281
ITAE	0.6623	0.6775	0.9205	0.6542
ITSE	0.0024	0.0042	0.08576	0.0014

In this part we have analyzed the position trajectory tracking errors in PMLM, various activation functions like sigmoid function (Sig F), hyperbolic tangent function (HTF), cosine function (CF), and hard limit function (HLF) has been evaluated using metrics like root mean square error (RMSE), integral absolute error (IAE), integral time absolute error (ITAE), and integral time square error (ITSE). As it can be seen that the sigmoid function excelled in minimizing steady state errors (lowest RMSE), whereas the hard limit function was most effective in reducing the overall error magnitude over time (lowest IAE). Both these functions showed comparable efficacy in minimizing error over time (ITAE), highlighting their suitability for applications where prolonged accuracy is crucial. The hard limit function also stood out in minimizing larger errors (lowest ITSE), critical for precision-demanding applications. The investigation of our proposed control highlights the significant impact of activation function choice on error dynamics in PMLM systems, emphasizing the need for careful selection based on specific application requirements.

## Conclusion

In this study we have proposed a sliding mode adaptive control law with predefined CT for PMLM motor with parameter perturbation, compound interference, perturbed state, and input saturated control constraints. This sliding mode control law not only ensure the position tracking error of PMLM motor remains within the preset performance function but also guarantees the boundness of the speed tracking error and the control input satisfies the preset bounded requirements. Furthermore, it also confirms that the motor position tracking accuracy reaches to the order of micro level within this predefined time. Consequently, the proposed PTCSMAC has achieved the higher precision balance between the motor displacement and tracking speed. The proposed adaptive control law has the advantages which only needs the trajectory tracking error information and its derivative but does not need any other information of the controlled object. Therefore, it belongs to an adaptive controller, which effectively improves the robustness and broaden application range of the proposed control. Besides this, it can be used for high-precision trajectory tracking for the other second-order nonlinear systems. The integration of fuzzy, RBF neuroadaptive, reinforcement learning (RL) and other artificial intelligent techniques with this predefined time sliding mode control can be considered in future work (“Supplementary information”).

### Supplementary Information

Supplementary Information.Appendix A

## Data Availability

All the data are included within the article. They can be provided on demand from the corresponding author.
