# Supplementary material for: Design and analysis of ELM-based predefined time sliding mode adaptive controller for PMLM position control under physical constraints
Source: Sci Rep. 2024 Mar 5;14:5408. doi: 10.1038/s41598-024-55444-4 (PMC11322532; doi:10.1038/s41598-024-55444-4)
Supplement: Supplementary file 1 — Supplementary Information.Appendix A [file 41598_2024_55444_MOESM1_ESM.docx]

**Appendix A:**

| **Notation** | **Physical significance** |
| --- | --- |
| $x_{1}(t)\text{,}x_{2}(t)$ | PMLM's location and speed |
| $y(t)$ | System’s output |
| $u(t)$ | Input (v) |
| $u_{M}$ | Maximum input (v) |
| $R$ | Resistance |
| $m$ | Mass of motor |
| $k_{f}$ | Constant (force) |
| $k_{e}$ | Back EMF |
| $F_{\text{friction}}(t)$ | Friction force |
| $F_{\text{ripple}}(t)$ | Ripple force |
| $F_{L}(t)$ | Load |
| $f_{v}$ | Viscous friction coefficient |
| $x_{2s}$ | Parameters for lubrication |
| $f_{c}$ | Coulomb friction’s minimum value |
| $f_{s}$ | Static friction |
| $\omega_{i}$,$A_{i}$ | Angular velocity and ripple force’s amplitude |
| $M,N,d$ | System’s intermediate variable |
| $y_{\text{d}}(t)$ | Desired trajectory |
| $e(t)$ | Trajectory error |
| $\rho_{0}$ | Performance Functions’ initials |
| $\rho_{\infty}$ | Steady state value of Pre-set Performance Functions |
| $\alpha$ | Convergence speed of Preset Performance Functions |
| $F_{1}(t)$ | Preset Performance Functions |
| $v(t)$ | Unconstrained input |
| $g(v(t))$ | Approximate function of bounded input |
| $\Delta u(t)$ | Approximate error of bounded input |
| $\xi$ | Intermediate variable of performance function |
| $S$ | Sliding mode surface |
| $T_{s1}$,$T_{s2}$ | Pre-defined time |
| $q$ | Sliding mode surface’s parameters |
| $t_{s}$ | Time of for convergence |
| $\vartheta(\xi)$ | 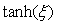 |
| $V(t)$ | Lyapunov function |
| $p,a,b$ | Lyapunov function’s parameters |
| $Q$ | Uncertainties of system |
| $\hat{Q}$ | Estimate of system uncertainties |
| $I$ | Identify matrix of appropriate dimension |
| $\mathbf{z}_{i}$ | Input of ELM |
| $\tilde{N}$ | Number of nodes |
| $\mathbf{a}_{i}$ | Connect weight vectors of ELM |
| $b_{i}$ | Node Threshold of ELM |
| $\mathbf{w}_{i}$ | External weight of ELM |
| $\hat{\mathbf{w}}_{i}$ | External weight estimate value of ELM |
| $\mathbf{w}^{*}$ | Optimal external weight value of ELM |
| $\tilde{\mathbf{w}}$ | Error of external weight value |
| $K_{1},K_{2},\kappa_{1},\kappa_{2},a^{*},b^{*}$ | Intermediate variable of stability proof |
| $\theta$ | 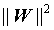 |
| $\hat{\theta}$ | Estimation of 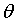 |
| $\tilde{\theta}$ | Estimate error of 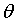 |
| $l_{\theta},b_{\theta}$ | Upper and lower bound (respectively) for 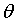 |
